# Flexible Formation of Nanoparticles: Selectively Self-Assembling with Glycoclusters to Form Nano-Photosensitizers for Multipurpose Bioimaging and Photodynamic Therapy

**DOI:** 10.3390/molecules30061274

**Published:** 2025-03-12

**Authors:** Kai-Li He, Wen-Jia Li, Yu Hu, Lu-Lu Sun, Lei Dong, Jing Xing, Jin Gong, Xiaoming Gong, Hai-Hao Han

**Affiliations:** 1 School of Pharmacy, Shandong Second Medical University, Weifang 261053, China; hekaili2023@163.com (K.-L.H.); 13893624832@163.com (Y.H.); xjing1790@163.com (J.X.); 2 Shandong Laboratory of Yantai Drug Discovery, Bohai Rim Advanced Research Institute for Drug Discovery, Yantai 264117, China; liwenjia@simm.ac.cn (W.-J.L.); llsun@baridd.ac.cn (L.-L.S.); 3 Molecular Imaging Center, Stake Key Laboratory of Chemical Biology, Shanghai Institute of Materia Medica, Chinese Academy of Sciences, Shanghai 201203, China; 4 University of Chinese Academy of Sciences, Beijing 100049, China; 5 Comprehensive Technical Service Center of Weifang Customs, Weifang 261041, China

**Keywords:** TPE-glycocluster, self-assembly, biological uptake, photodynamic therapy

## Abstract

The smart construction of nano-photosensitizers (PSs) is significant for multipurpose applications, such as bioimaging, efficient photodynamic anti-tumor or anti-bacterial studies. This work reports a flexible self-assembling strategy for the construction of nano-PSs, in which PSs spontaneously form amorphous aggregates for killing bacteria, or self-assemble with tetraphenylethene (TPE) based glycoclusters (**TPE-Glc_4_**) to construct glyco-dots for cell imaging and photodynamic anti-tumor studies. Tricyanofuran (TCF) and TPE units were bridged with furan or thiophene moiety to construct two PSs (**1** and **2**) with NIR fluorescence in monomers, and a performance of the aggregation-induced generation of reactive oxygen species (AIG-ROS) in an aggregated state. Compared to the large amorphous aggregates (**2-a**), TPE-based glycoclusters encapsulated with PS form glyco-dots (**2-Glc**) that exhibit a smaller and more homogeneous hydrated size of approximately 40 nm, as well as enhanced water-solubility and biocompatibility. TPE-glycoclusters facilitate the cellular uptake of **2** into HepG2 cells, therefore enhancing the NIR fluorescence imaging signal and photodynamic therapy. Meanwhile, **2-a** exhibits satisfied phototoxicity against *Escherichia coli*. This work highlights the flexible self-assembly of nano-PSs for multifunctional bioapplications.

## 1. Introduction

Photodynamic therapy (PDT), hailed as a pioneering treatment method, has attracted considerable attention due to its wide array of applications [1,2], including fighting cancer, treating microbial infections, and acting as a pesticide in agriculture [3,4,5]. Photosensitizers (PSs) are integral and multifaceted elements in PDT, are able to stimulate oxygen to generate reactive oxygen species (ROS) such as singlet oxygen (^1^O_2_), superoxide (·O_2_^−^), or hydroxyl radicals (·OH), as well as provide fluorescence signals for bioimaging and diagnosis [6,7,8,9,10]. Recently, the advancement of PSs has been speeding up at an impressive pace. A variety of small organic PSs, such as porphyrins, dibromo boron dipyrromethene (BODIPY), and phthalocyanines, have shown high effectiveness in PDT [11,12]. The fabrication of nanoparticles via supramolecular assembly represents a sophisticated approach to engineer nano-PSs that possess tailored ROS production or biological functions [13,14]. Some nanoparticles, constructed from an electron donor and acceptor, facilitate the electron transfer process to specifically generate Type-I ROS (·O_2_^−^, ·OH) [15]. Tang’s group has presented a series of PSs with the properties of aggregation-induced generation of reactive oxygen species (AIG-ROS), which not only boosts PDT effectiveness but also provides fluorescence signals within the nanoparticles [16,17].

Despite numerous PSs being reported, their broader biological application still faces numerous hurdles. A pressing challenge is the hydrophobic structure that causes excessive intermolecular aggregation, which in turn leads to poor biocompatibility and diminished cellular uptake. Integrating hydrophilic and bioactive components into PSs, like polyethylene glycol (PEG) chains and peptide sequences, markedly improves their biocompatibility and internalized efficiency in cells through endocytosis [18,19,20]. However, this method requires additional functional sites on the PS architecture to accommodate these modifications. An alternative sophisticated approach is the self-assembly of glycoclusters with PSs into nanoparticles, which has demonstrated an enhancement in the biological properties of PSs [21]. In our previous research, we utilized glycoclusters to facilitate the self-assembly of fluorescent sensors, thereby enhancing their aqueous disperse and detection sensitivity both in solution and cells [22,23]. Therefore, we suggested that glycoclusters might also effectively augment the biological internalization of PSs.

**Figure 1:**
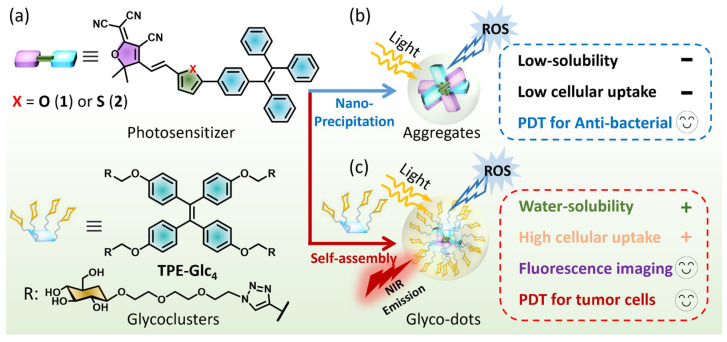
(**a**) Structures of TCF-based PSs **1**, **2,** and TPE-based glycoclusters. (**b**) PSs spontaneously formed amorphous aggregates through nano-precipitation for killing bacteria under light irradiation. (**c**) PSs self-assembled with or TPE-glycoclusters to form glyco-dots for enhanced NIR imaging and PDT of tumor cells.

In this study, we develop a flexible self-assembly strategy, in which tricyanofuran (TCF)-based PSs spontaneously form amorphous aggregates for anti-bacteria or self-assemble with glucosyl TPE-glycoclusters to construct glyco-dots for near-infrared (NIR) imaging and photodynamic anti-tumor studies (Figure 1). Two PSs are composed of an electron acceptor (TCF) and electron donor (TPE), which are bridged by furan (**1**) or thiophene (**2**) moieties (Figure 1a). The ICT-based PSs display NIR fluorescence in monomers, and an AIG-ROS capability to sensitize oxygen to singlet oxygen (^1^O_2_), superoxide radicals (·O_2_^−^), and hydroxyl radicals (·OH) upon the formation of aggregates. Compared with amorphous aggregates with a large, hydrated size and sheet-like morphology, the glyco-dots possess a hydrated diameter of approximately 40 nm and homogenous morphologies with improved water-solubility and biocompatibility. **TPE-Glc_4_** facilities the cellular uptake of PS **2** into HepG2 cells, thereby enhancing both NIR fluorescence imaging and photodynamic anti-tumor efficacy, whereas amorphous aggregates **2-a** exhibit outstanding phototoxicity for killing *E. coli* under light irradiation (Figure 1b,c).

## 2. Results and Discussion

### 2.1. Structure and Photophysical Properties

We designed and synthesized two tricyanofuran (TCF)-based PSs featuring an electronic “pull–push” architecture (Figure 1a and Scheme S1). The tetraphenylethene (TPE) segment, recognized for its twisted structure and electron-rich nature, efficiently inhibits the aggregation-caused quenching of ROS. We chose either furan (**1**) or thiophene (**2**) units to build the “D-π-A” framework for extending intramolecular π-conjugation. Our previous research has detailed the synthesis of glucosyl TPE-based glycoclusters (**TPE-Glc_4_**), which possess a twisted hydrophobic core and four glucosyl TEG chains, capable of encapsulating PSs to create glyco-dots (Scheme S1) [24]. All characterization data, including ^1^H NMR, ^13^C NMR, and high-resolution mass spectrometry (HRMS), have been provided in the Supplementary Materials.

**Figure 2:**
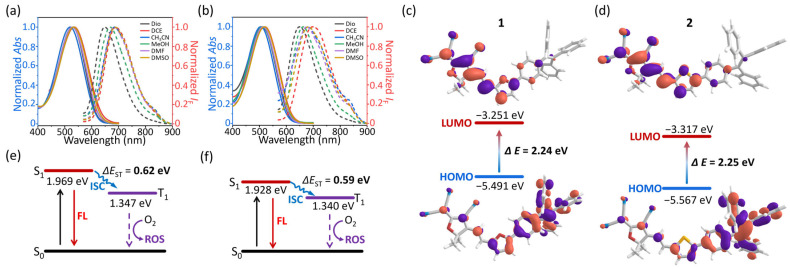
Normalized absorption (solid line) and fluorescence spectra (dashed line) of (**a**) **1** and (**b**) **2** in different organic solvents (c = 5 μM, λ_ex_ = 520 nm). Density functional theory (DFT) calculated the HOMO and LUMOs of (**c**) **1** and (**d**) **2**. DFT calculated the excited energy of S_1_ and T_1_ state of (**e**) **1** and (**f**) **2**.

We initially discussed their photophysical characteristics across a range of organic solvents, including 1,4-dioxane (Dio), 1,2-chloroethane (DCE), CH_3_CN, MeOH, DMF, and DMSO (Table S1). The TCF-based PSs demonstrated a single, broad absorption band that ranged from 400 to 700 nm, with peak absorption at 534 nm (*ε* = 4.2 × 10^4^ M^−1^ cm^−1^) for **1** and at 520 nm (*ε* = 5.9 × 10^4^ M^−1^ cm^−1^) for **2** in DMSO (Figure 2a,b). The “D-π-A” backbone of PSs confers their fluorescence emissions in near-infrared (NIR) region, marked by a broad emission band stretching from 600 to 900 nm (Figure S1). The recorded fluorescence peaks for **1** and **2** were at 689 nm (*Φ*_F_ = 1.9%) and 685 nm (*Φ*_F_ = 1.6%) in DMSO, respectively (Table S1). The optical properties of PSs exhibit pronounced characters of intramolecular charge transfer (ICT).

To further demonstrate our hypothesis, functional theory (DFT) calculations were utilized to determine the highest occupied molecular orbitals (HOMOs), the lowest unoccupied molecular orbitals (LUMOs), and the excited energy of the S_1_ state for PSs. It was observed that the electronic density on the HOMOs was predominantly localized on the TPE and bridge units, meanwhile the LUMOs exhibited nearly uniform delocalization across the TCF and bridge segments (Figure 2c,d and Figure S2). The significant electron separation illustrated the ICT effect, which probably influenced the photophysical properties of **1** and **2**. The distributions of electrons and holes within the S_1_ state revealed that electrons were primarily located on the TCF units and the π-backbone, whereas holes were almost exclusively concentrated on the TPE parts (Figure S3) [25,26,27,28]. These observations further corroborate that the ICT effect is the main influencing factor for mastering the optical spectra of PSs. In addition, the excitation energies of the S_1_ state were determined to be 1.969 eV for **1** and 1.928 eV for **2**, which aligns with our photophysical analysis (Figure 2e,f).

### 2.2. Formation of Aggregates and Glyco-Dots

**Figure 3:**
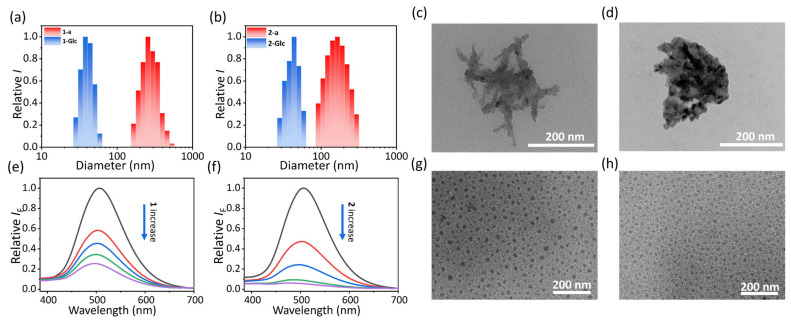
Dynamic light scatting measurements (DLS) of the average hydrated size of amorphous aggregates (blue) and glyco-dots (red) for (**a**) **1** and (**b**) **2**. Transmission electron microscopy (TEM) images of (**c**) **1-a** and (**d**) **2-a**. Relative fluorescence variation in TPE-glycoclusters upon addition of (**e**) **1** and (**f**) **2** in PBS buffer. TEM images of (**g**) **1-Glc** and (**h**) **2-Glc**.

Next, we investigated the self-assembly of two PSs in PBS buffer (0.01 M, pH 7.4). For the hydrophobic structures, both **1** and **2** rapidly formed the corresponding amorphous aggregates **1-a** and **2-a** (Figure 1b). Compared with the monomer in DMSO, **1-a** and **2-a** have blue-shifted, widened, and impaired absorption bands, whose maximum wavelength were located at 525 nm (*ε* = 2.12 × 10^4^ M^−1^ cm^−1^) for **1-a** and 510 nm (*ε* = 2.09 × 10^4^ M^−1^ cm^−1^) for **2-a** (Figure S4). However, the fluorescence of two aggregates have been red-shifted to 716 nm and 720 nm, respectively, with dim brightness (Figure S5). Dynamic light scattering (DLS) and a transmission electron microscope (TEM) were employed to determine the hydrate sizes and morphologies of the aggregates. A broad size distribution with a large average hydrated diameter (ca. 256 nm) of **1-a** was measured (Figure 3a, red bar), followed by the irregular, sheet-like morphology from TEM image (Figure 3c). Both **2-a** and **1-a** showed a similar hydrated size distribution (ca. 197 nm) and morphology (Figure 3b,d). The large volume of aggregates represented poor water-stability, leading to slow precipitation in the aqueous environment.

To enhance the water stability of PSs, **TPE-Glc_4_** was self-assembled with PSs to accomplish the formation of glyco-dots **1-Glc** and **2-Glc** (Figure 1c). The amphiphilic **TPE-Glc_4_** is supposed to enable the hydrophobic, twisted TPE core to encapsulate PSs, while its hydrophilic and pliable PEG chains and glucosides promote interaction with water. Evidence of self-assembly was observed through the progressive emission decrease in TPE**-Glc_4_** at 505 nm upon increasing concentrations of **1** or **2** (Figure 3e,f). In addition, the absorption bands of PS were found to closely overlap with the emission band of **TPE-Glc_4_** (Figure S6), suggesting a potential intermolecular Förster resonance energy transfer (FRET) within the glyco-dots, which could account for the observed fluorescence quenching of **TPE-Glc_4_.** Self-assembly with **TPE-Glc_4_** did not clearly alter the absorption band of glyco-dots, with maxima located at 525 nm (*ε* = 2.14 × 10^4^ M^−1^ cm^−1^) for **1-Glc** and 510 nm (*ε* = 2.18 × 10^4^ M^−1^ cm^−1^) for **2-Glc** (Figure S4). The fluorescence spectra of glyco-dots showed a modest enhancement compared to aggregates, indicating that the PSs have been well encapsulated within the hydrophobic cavities of the glyco-dots (Figure S5). DLS revealed that the average hydrodynamic size of the glyco-dots was suddenly reduced to ca. 40 nm for **1-Glc** and 42 nm for **2-Glc** (Figure 3a,b, blue bar). TEM images confirmed that both glyco-dots presented homogenous, compact and granular morphologies (Figure 3g,h). Larger nanoparticles sizes were found in DLS than in TEM images. We suggest that the glycoside and unfolded TEG chains of glyco-dots interacted with water, resulting in the observation of their larger hydrated size. Additionally, the glyco-dots exhibited improved water stability at room temperature, maintaining their integrity over a 6-day period (Figure S7). These findings robustly substantiate the hypothesis that **TPE-Glc_4_** plays a crucial role in facilitating the dispersion of PSs in an aqueous environment.

### 2.3. ROS Generation of Aggregates and Glyco-Dots

**Figure 4:**
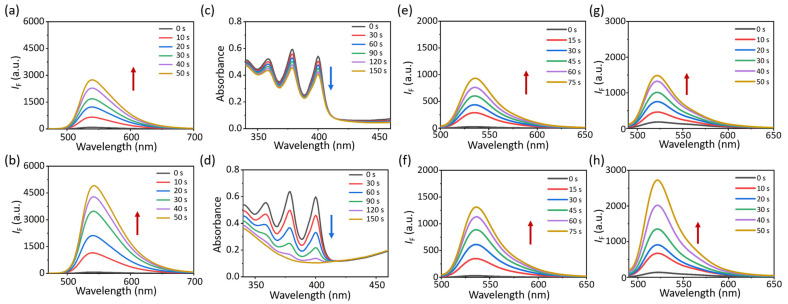
Fluorescence spectra of DCFH in PBS dispersion of (**a**) **1-Glc** and (**b**) **2-Glc** (c = 10 μM) under light for 50 s (interval 10 s, λ_ex_ (DCFH) = 470 nm). Absorption spectra of ABDA in PBS dispersion of (**c**) **1-Glc** and (**d**) **2-Glc** (c = 10 μM) under light for 150 s (interval 30 s, *p* = 10 mW cm^−2^). Fluorescence spectra of DHR123 in PBS dispersion of (**e**) **1-Glc** and (**f**) **2-Glc** (c = 10 μM) under light for 75 s (interval 15 s, λ_ex_ (DHR123) = 470 nm). Fluorescence spectra of HPF in PBS dispersion of (**g**) **1-Glc** and (**h**) **2-Glc** (c = 10 μM) under light for 50 s (interval 10 s, λ_ex_ (HPF) = 470 nm, 590 nm LED light, *p* = 10 mW cm^−2^). Red and blue arrows represent the intensive increase and decrease, respectively.

We assessed the ROS generation capability of two PSs upon exposure to light irradiation. DFT was employed to calculate energy gaps between the singlet (S_1_) and triplet (T_1_) states for the two compounds, which were determined to be 0.62 eV for **1** and 0.59 eV for **2**, respectively (Figure 2e,f). The low Δ_EST_ might facilitate the intersystem crossing (ISC) of excited electrons. Then, 2′,7′-dichlorofluorescein (DCFH) was utilized to monitor the ROS generation of **1** and **2** in monomers, amorphous aggregates, and glyco-dots states. Under 590 nm light (*p* = 10 mW cm^−2^), neither monomeric **1** nor **2** in DMF could “trigger” the DCFH fluorescence (Figure S8). Nevertheless, we observed the fluorescence enhancement of DCFH in both the PBS dispersion of aggregates (Figure S9) and glyco-dots (Figure 4a,b, Figures S10 and S11a) as the light irradiation time prolonged, signifying the outstanding capability of the two PSs for the aggregation-induced generation of ROS (AIG-ROS) [29,30]. To further illuminate the ROS generation, 9,10-anthracenediyl-bis(methylene)dimalonic acid (ABDA) was enlisted to monitor the singlet oxygen generation. After 150 s of light activation, the ABDA absorption reduction of **2-Glc** was more rapid than that of **1-Glc**, suggesting that **2-Glc** possesses a superior ^1^O_2_ generation ability through a Type-II mechanism (Figure 4c,d, Figures S11b and S12). The similar ABDA decrease trends of **1-a** and **2-a** were monitored in the same way (Figure S13).

Beyond ^1^O_2_, we questioned if other types of ROS were also produced. Dihydrorhodamine 123 (DHR123) was utilized to explore the superoxide radical (O_2_^−^) generation. After turning on the light, the fluorescence enhancement at 530 nm could be distinctly observed within 75 s (Figure 4e,f, Figures S11c and S14). To further affirm the ·O_2_^−^ generation, we used dihydroethidium (DHE) to detect the ·O_2_^−^, and using the fluorescence at 600 nm can begin after capturing ·O_2_^−^. In a dark environment, the DHE kept a quenched fluorescence in aqueous solution. While exposing under 590 nm light irradiation, the fluorescence enhancement at 600 nm of DHE can be clearly found within 5 min (Figures S15 and S16). These results indicated the glyco-dots evidently sensitized oxygen to ·O_2_^−^. Moreover, the fluorescence increase in hydroxyphenyl fluorescein (HPF, a commercial ·OH probe) at 520 nm was measured in the PBS dispersion of glyco-dots exposed to light, demonstrating that another type of Type-I ROS, the hydroxyl radicals, were generated by glyco-dots (Figure 4g,h, Figures S11d and S17). Similarly, the amorphous aggregates **1-a** and **2-a** that could generate Type-I ROS of ·O_2_^−^, and ·OH under light irradiation were verified (Figures S18–S20). This confirmed that the generation of ROS was initiated by intermolecular aggregation, rather than the self-assembly with glycoclusters. In addition, **2-Glc** exhibited better photo-stability than **1-Glc**, with only a marginal decrease in absorption after being exposed to 590 nm light irradiation (*p* = 30 mW cm^−2^) for 50 min (Figure S21).

### 2.4. NIR Cell Imaging and Photodynamic Therapy in HepG2 Cells

**Figure 5:**
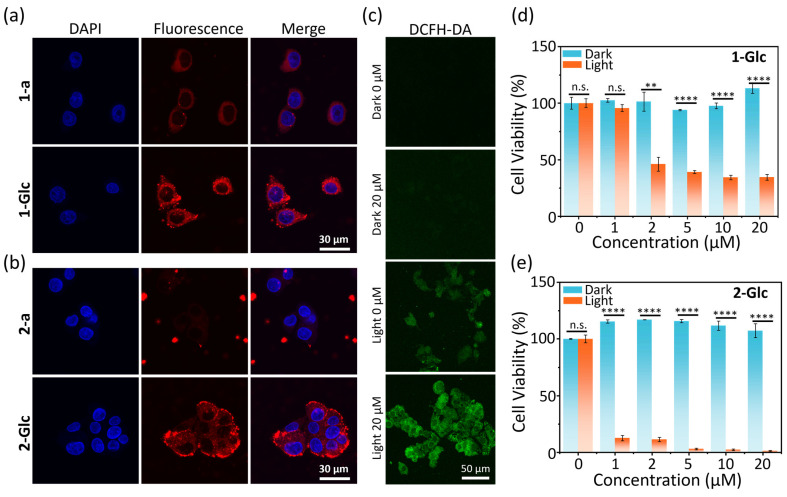
Laser confocal microscope imaging of HepG2 cells incubating with aggregates or glyco-dots of (**a**) **1** and (**b**) **2**. (**c**) DCFH-DA sensing the intracellular ROS generation after treating with **2-Glc** and irradiating with or without white light. Cell viability of HepG2 cells stained with different concentrations of (**d**) **1-Glc** and (**e**) **2-Glc**, and then irradiated with (yellow bar) or without (blue bar) white light (*p* = 30 mW cm^−2^, 2 h). Blue channel for DAPI: Ex/Em = 405/430–470 nm. Red channel for glyco-dots: Ex/Em = 561/600–700 nm. Green channel for DCFH-DA: Ex/Em = 488/500–600 nm. Error bars represent S. D. (n = 3), n.s. no significance, ** *p* < 0.01, **** *p* < 0.0001.

Building on the impressive ROS generation capabilities of glyco-dots in solution, we explored their potential for fluorescence imaging and PDT within cellular contexts. Next, we assessed the cellular uptake of both aggregates (**1-a** and **2-a**) and glyco-dots (**1-Glc** and **2-Glc**). Incubation with HepG2 cells followed by laser confocal microscopy revealed a marked increase in NIR fluorescence in cells treated with glyco-dots, contrasting with the weaker signals observed from aggregates lacking **TPE-Glc_4_** (Figure 5a,b). A quantitative analysis of intracellular fluorescence intensity confirmed that **2-Glc** led to a two-fold increase in fluorescence signal compared to its individual incubation (**2-a**), underscoring the role of **TPE-Glc_4_** in facilitating the cellular uptake of PSs (Figure S22). We hypothesize that the peripheral glucosides on the glyco-dots significantly enhance their cellular affinity, thereby promoting the cellular uptake of PSs. This strategic modification could pave the way for improving the effectiveness of phototherapeutic agents in cancer treatment.

Encouraged by the improved cellular uptake of glyco-dots, we investigated their potential in PDT for HepG2 cells. Various concentrations (0–20 μM) of **1-Glc** or **2-Glc** were cultured with HepG2 cells before being placed in a dark environment or exposing under white LED light (*p* = 30 mW cm^−2^) for 2 h. Cell viability showed unaffected in the dark, but under light irradiation, it decreased progressively with increasing concentrations of glyco-dots (Figure 5d,e). Notably, **2-Glc** exhibited superior phototoxicity compared with **1-Glc**, which could decrease the cell viability to <20% even at a low concentration of 1 μM (Figure 5e). From living-dead cells co-staining images, it was evident that only the group treated with **2-Glc** and exposed to light irradiation exhibited the red fluorescence of propidium iodide (PI) from dead cells, in contrast to the green fluorescence of Calcein AM from living cells (Figure S23). Intracellular ROS imaging using DCFH-DA confirmed that **2-Glc** sensitized oxygen to generate ROS under light irradiation within cells, leading to cell death (Figure 5c and Figure S24).

### 2.5. Photodynamic Therapy for Anti-Bacterial Studies

**Figure 6:**
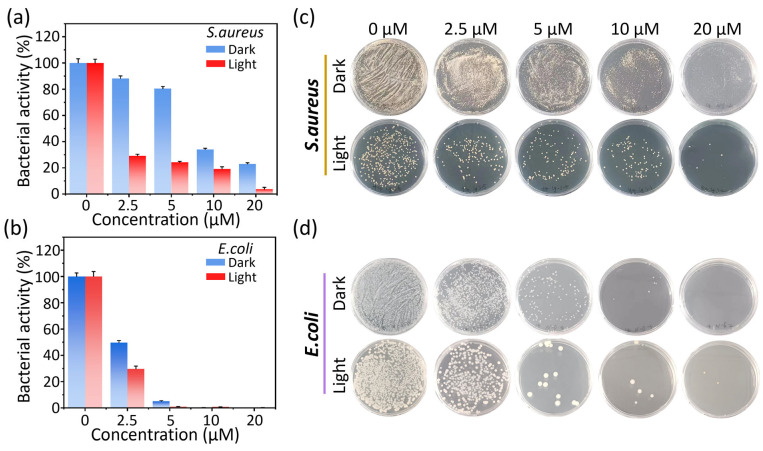
Statistical relative bacterial activities of (**a**) *S. aureus* and (**b**) *E. coli* incubated with different concentrations of **2-a** (0–20 μM) in dark (blue bar) or illuminated (red bar) conditions. Plate images of (**c**) *S. aureus* and (**d**) *E. coli* incubated with different concentrations of **2-a** (0–20 μM) in (**c**) dark or (**d**) illuminated conditions (*p* = 30 mW cm^−2^, 2 h).

It is well-known that bacteria possess more a rigid cytoderm than mammalian cells, thus hindering PSs from penetrating and effectively killing bacteria. Encouraged by the excellent ROS generation of **2-a** and **2-Glc**, we evaluated photodynamic anti-bacterial studies on Staphylococcus aureus (*S. aureus*, a Gram-positive bacterium) and *Escherichia coli* (*E. coli*, a Gram-negative bacterium). The relative activity of *S. aureus* and *E. coli* was investigated after incubation with **2-a** both in the dark and under light exposure (*p* = 30 mW cm^−2^), respectively. Compared with *S. aureus*, **2-a** exhibited a higher toxicity against *E. coli* in a dark environment. We speculated that the dark toxicity of **2-a** for bacteria might be embedded in bacterial cell membranes through hydrophobic interactions or electrostatic interactions, disrupting membrane integrity and function, leading to the leakage of substances within the bacterial cell and affecting the normal physiological functions of the bacteria [31,32]. On this basis, we turned on the white LED light, and observed that the bacterial activities were significantly restrained with a minimum inhibitory concentration (MIC90) of 20 μM for *S. aureus* and 5 μM for *E. coli*, respectively (Figure 6). We subsequently evaluated the phototoxicity of **2-Glc** against *E. coli* under light irradiation (*p* = 30 mW cm^−2^). The raised bacteria activity of *E. coli* indicated that encapsulation of TPE-glycoclusters did not improve the photodynamic anti-bacteria, which was contrary to the PDT effect for anti-tumor studies. We supposed that TPE-glycoclusters might hinder the interaction between PSs and bacteria, thus impairing both dark and photo toxicity for anti-bacteria (Figure S25).

## 3. Materials and Methods

### 3.1. Synthesis

Synthesis procedure of compound **1**: All reagents for synthesis commercially available were used without further purification (Anhui Senrise Technologies Co., Ltd., Hefei, China). To a solution of **1-S1** (245 mg, 0.574 mmol, 1 eq.) and 2-(3-cyano-4,5,5-trimethylfuran-2(*5H*)-ylidene)malononitrile (TCF, 137 mg, 0.689 mmol, 1.2 eq.) in CH_3_CN (20 mL), we added piperidine (0.3 mL) and AcOH (0.3 mL). The mixture was refluxed until TCL monitored the disappearance of the starting materials. The reaction was diluted with EtOAc (50 mL) and washed with HCl aqueous solution (2 M, 40 mL) and brine (40 mL×3). The combined organic layer was dried (Na_2_SO_4_), concentrated, and purified with silica gel column chromatography (PE:EtOAc = 3:1) to obtain compound **1** (231 mg, 66%) as a dark purple powder. ^1^H NMR (600 MHz, CDCl_3_): δ (ppm) 7.54 (d, *J* = 8.5 Hz, 2H), 7.50 (d, *J* = 15.8 Hz, 1H), 7.09–7.17 (m, 12H), 7.09–7.06 (m, 2H), 7.05–7.00 (m, 4H), 6.83 (d, *J* = 3.8 Hz, 1H), 6.78 (d, *J* = 15.8 Hz, 1H), 1.74 (s, 6H). ^13^C NMR (151 MHz, CDCl_3_): δ (ppm) 176.0, 173.3, 161.5, 160.8, 160.6, 150.7, 146.4, 143.7, 143.6, 143.5, 142.7, 140.3, 132.5, 131.71, 131.69, 131.6, 128.4, 128.3, 128.2, 128.1, 127.2, 127.1, 124.9, 112.4, 111.6, 111.1, 110.4, 97.5, 57.0, 26.7. HR-ESI-MS *m*/*z*: calcd. for C_42_H_29_N_3_O_2_Na^+^ [M + Na]^+^ 630.2152, found 630.2142.

Synthesis procedure of compound **2**: To a solution of **2-S1** (300 mg, 0.677 mmol, 1 eq.) and TCF (162 mg, 0.813 mmol, 1.2 eq.) in CH_3_CN (20 mL), we added piperidine (0.3 mL) and AcOH (0.3 mL). The mixture was refluxed until TCL monitored the disappearance of starting materials. The reaction was diluted with EtOAc (50 mL) and washed with HCl aqueous solution (2 M, 40 mL) and brine (40 mL×3). The combined organic layer was dried (Na_2_SO_4_), concentrated, and purified with silica gel column chromatography (PE:EtOAc = 3:1) to obtain compound **2** (228 mg, 54%) as a dark purple powder. ^1^H NMR (600 MHz, DMSO-*d*_6_): *δ* (ppm) 7.80 (d, *J* = 15.8 Hz, 1H), 7.43 (d, *J* = 4.0 Hz, 1H), 7.40 (d, *J* = 8.2 Hz, 2H), 7.33 (d, *J* = 4.1 Hz, 1H), 7.15–7.13 (m, 6H), 7.12–7.10 (m, 4H), 7.10–7.05 (m, 3H), 7.06–7.00 (m, 4H), 6.65 (d, *J* = 15.9 Hz, 1H), 1.76 (s, 6H). ^13^C NMR (150 MHz, DMSO-*d*_6_): *δ* (ppm) 175.8, 173.4, 153.5, 146.1, 143.7, 143.6, 143.5, 142.7, 140.2, 139.8, 139.1, 137.1, 132.6, 131.69, 131.66, 131.6, 128.3, 128.2, 128.0, 127.2, 127.12, 127.08, 125.8, 125.5, 113.0, 112.2, 111.5, 111.0, 97.5, 57.3, 26.8. HR-ESI-MS *m*/*z*: calcd. for C_42_H_29_N_3_OSNa^+^ [M + Na]^+^ 646.1924, found 646.1918.

### 3.2. UV–Vis Absorption

The UV–Vis absorption spectra were measured at room temperature using a Hitachi UV-3900 spectrophotometer (Hitachi High-Technologies Co., Tokyo, Japan). All spectra were corrected for background intensities by subtracting the spectra of pure solvent measured under identical conditions. The absorbance of **1** and **2** (c = 5 μM) in monomer states were measured in various organic solvents. The relative absorption (*Abs*) was normalized by setting the maximum values of the spectra to 1, with all other values scaled proportionally within the [0, 1] range.

### 3.3. Fluorescence Spectroscopy

The fluorescence measurements were carried out at room temperature using Hitachi F-4600 spectrophotometer (Hitachi High-Technologies Co., Tokyo, Japan). The fluorescence emission of **1** and **2** (c = 5 μM) in the monomer state was measured in various organic solvents (λ_ex_ = 520 nm, slit width 5–10 nm, 700 V). The relative emission intensity (*I*_F_) were normalized by setting the maximum values of the spectra to 1, with all other values scaled proportionally within the [0, 1] range

### 3.4. Absolute Fluorescence Quantum Yield

Either compound **1** or **2** was diluted in DMSO with concentration of 5 μM. The absolute fluorescence quantum yield was determined by Hamamatsu Quantaurus-QY.

### 3.5. Theoretical Calculations

Gaussian 16 program was used to perform the density functional theory (DFT) calculations (b3lyp/6–31g(d,p)) of **1** and **2**. The high-performance computing server was provided by Beijing Super Cloud Computing Center, Beijing, China.

### 3.6. Formation of Amorphous Aggregates

Either compound **1** or **2** (1 mmol) was well-dissolved in DMSO (20 μL). To the resulting solution we added 9980 μL deionized (DI) water or PBS buffer (pH 7.4, 1 mM), and this was then dispersed under ultrasound in ice bath for 10 min to form the amorphous aggregates (**1-a** or **2-a**, 100 μM). The resulting dispersion in water or PBS buffer was diluted to a low concentration for spectral measurements.

### 3.7. Formation of Glyco-Dots

Either compound **1** or **2** (1 mmol) was well-dissolved in DMSO (20 μL). **TPE-Glc_4_** (10 mmol) was well-diluted in H_2_O or PBS buffer (80 μL). The DMSO solution of **1** or **2** was mixed with **TPE-Glc_4_**, and then we added 9900 μL deionized (DI) water or PBS buffer. The resulting solution was sufficiently dispersed under ultrasound in an ice bath for 10 min to form the glycol-dots (**1-Glc** or **2-Glc**, 100 μM). The glyco-dots dispersion in water or PBS buffer was diluted to low concentration for spectral measurements.

### 3.8. Cell Lines and Cell Culture

Hepatocellular cell line HepG2 cells were obtained from the American Type Culture Collection (ATCC HB-8065, Alexandria, MN, USA). The tested HepG2 cells were cultured in high glucose DMEM supplemented with 10% fetal bovine serum (FBS) and 0.2% penicillin–streptomycin with 5% CO_2_ at 37 °C.

### 3.9. In Vitro Dark/Light Cytotoxicity

HepG2 cells were seeded in 96-well plates (8 × 10^3^ cells/well) and incubated for 24 h under normoxia. Then, the medium was replaced with 100 μL of DMEM supplemented with 1% DMSO containing different concentrations (0–20 μM) of glycol-dots. After incubation for another 4 h, the cells were washed three times with PBS, infused with fresh medium, and illuminated by a white LED light (30 mW cm^−2^) for 2 h. After that, the cell viability was examined by cell counting kit-8 (CCK-8) assays. All experiments were repeated three times with representative data shown. Moreover, the dark toxicity of glycol-dots was also analyzed by the above procedure except the illumination was eliminated.

### 3.10. Cell Uptake and Imaging

HepG2 cells were seeded on 24-well plates (3 × 10^4^ cells/well) cultured in DMEM medium containing 10% FBS under normoxia for 24 h. Then, the medium was replaced with culture medium with aggregates (**1-a** or **2-a**, 10 μM) or glycol-dots (**1-Glc** or **2-Glc**, 10 μM), and then further incubated for 2 h. Subsequently, the cells were washed three times with PBS and imaged by confocal microscopy.

### 3.11. Bacterial Toxicity Assay

An equal volume of bacterial solution (100 μL, ca. 1 × 10^5^ CFU mL^−1^) was added to the diluted PS solution in a 48 well plate, and then the 48 well plate was incubated at 37 °C for 1 h. The dispersion of **2-a** and **2-Glc** was added to the bacterial solution with different final concentrations as 0, 4, 8, 12, 16 μM and incubated for 80 min. After irradiating under white LED light for 30 min (30 mW cm^−2^), 100 μL bacterial solution was coated on solid LB medium plate. After incubation at 37 °C for 10 h, the number of bacterial single colonies on the solid LB medium plate was recorded. The minimum inhibitory concentration 90 (MIC_90_) was determined as the lowest concentration of PSs at which we observed that the bacterial relative activity was inhibited to >90%. The dark toxicity assay of amorphous aggregates and glycol-nanoparticles were analyzed in the same experimental conditions but without light irradiation.

## 4. Conclusions

In summary, we have successfully developed two TCF-based PSs **1** and **2** with NIR fluorescence in monomer. Two PSs could flexibly select to form amorphous aggregates (**1-a** and **2-a**) with large and sheet-like morphology, or self-assemble with TPE-glycoclusters (**TPE-Glc_4_**) to construct the small and nubby glyco-dots (**1-Glc** and **2-Glc**) in an aqueous environment. Both the aggregates and glyco-dots exhibited outstanding AIG-ROS ability, allowing us to sensitize oxygen to multiple kinds of ROS (^1^O_2_, ·O_2_^−^, and ·OH) through Type-I or Type-II processes. For glyco-dots, **TPE-Glc_4_** efficiently facilitated the delivery of **2** into HepG2 cells, thereby enhancing the intracellular fluorescence signal and PDT effect for anti-tumor studies. In addition, amorphous aggregates **2-a** exhibited outstanding phototoxicity for killing *E. coli* under light irradiation. This work highlights the novel formation strategy of PSs, that flexibly self-assembled with or without glycoclusters to realize the multifunctional fluorescence imaging and photodynamic applications of anti-tumor and anti-bacteria studies.

## Data Availability

Data are contained within the article and Supplementary Materials.

## Supplementary Materials

The following supporting information can be downloaded at https://www.mdpi.com/article/10.3390/molecules30061274/s1. Scheme S1: Synthesis procedures of PSs 1 and 2; Table S1: Photophysical properties of 1 and 2 in various solvents; Figure S1: Absorption spectra and molar extinction coeffcient (*ε*) of 1 and 2 in different organic solvents; Figure S2: Density functional theory (DFT) calculated the molecular optimal conformation of 1 and 2; Figure S3: DFT calculated the electron–hole distribution of 1 and 2 at singlet excited (S_1_) state; Figure S4: Normalized absorption of compounds 1 and 2 within monomer, amorphous aggregates and glycol-dots states; Figure S5: Relative fluorescence spectra of compound 1 and 2 within amorphous aggregates and glycol-dots states; Figure S6: Normalized absorption and fluorescence spectra of TPE-Glc_4_ compared with that of 1 and 2; Figure S7: Relative absorption spectra of 1-Glc and 2-Glc in PBS buffer at room temperaute for 6 d. (c) Corresponding relative absorbance variation at 520 nm of 1-Glc and 2-Glc in PBS buffer for 7 d. Figure S8: Fluorescence spectra of DCFH in DMF solution of (a) blank, (b) 1 and (c) 2 under 590 nm light irradiation for 50 s; Figure S9: Fluorescence spectra of DCFH in PBS of (a) blank, (b) 1-a and (b) 2-a under 590 nm light irradiation for 50 s; Figure S10: (a) Fluorescence spectra of TPE-Glc_4_ in PBS under 590 light irradiation for 50 s, (b) Fluorescence spectra of DCFH in PBS buffer without PSs under 590 nm light irradiation for 50 s, (c) Fluorescence spectra of DCFH in PBS dispersion of TPE-Glc_4_ under 590 nm light irradiation for 50 s; Figure S11: (a) Fluorescence variation in DCFH in PBS dispersion of 1-Glc and 2-Glc under light for 50 s, (b) Absorption variation in ABDA in PBS 1-Glc and 2-Glc under light for 150 s, (c) Fluorescence variation in DHR123 in PBS dispersion of 1-Glc and 2-Glc under light for 75 s, (d) Fluorescence variation in HPF in PBS dispersion of 1-Glc and 2-Glc under light for 50 s; Figure S12: (a) Absorption spectra of ABDA in PBS buffer without PSs under 590 nm light irradiation for 5 min, (b) Absorption spectra of ABDA in PBS dispersion of TPE-Glc_4_ under 590 nm light irradiation for 5 min; Figure S13: Absorption spectra of ABDA in PBS dispersion of (a) 1-a and (b) 2-a under 590 nm light irradiation for 150 s; Figure S14: (a) Fluorescence spectra of DHR123 in PBS buffer without PSs under 590 nm light irradiation for 75 s, (b) Fluorescence spectra of DHR123 in PBS dispersion of TPE-Glc_4_ under 590 nm light irradiation for 75 s; Figure S15: (a) Fluorescence spectra of DHE mixed with DNA in PBS buffer without PSs (blank sample) under 590 nm light irradiation for 5 min. (b) Fluorescence spectra of DHE mixed with DNA in PBS dispersion of **TPE-Glc_4_** under 590 nm light irradiation for 5 min; Figure S16: Fluorescence spectra of DHE mixed with DNA in PBS dispersion of (a) **1-Glc** and (b) **2-Glc** under light for 5 min. (c) Fluorescence variation in DHE mixed with DNA in PBS dispersion of **1-Glc** and **2-Glc** under light for 5 min; Figure S17: (a) Fluorescence spectra of HPF in PBS buffer without PSs under 590 nm light irradiation for 50 s, (b) Fluorescence spectra of HPF in PBS dispersion of TPE-Glc_4_ under 590 nm light irradiation for 50 s; Figure S18: Fluorescence spectra of DHR123 in PBS dispersion of (a) 1-a and (b) 2-a under 590 nm light irradiation for 75 s; Figure S19: Fluorescence spectra of DHE mixed with DNA in PBS dispersion of (a) **1-a** and (b) **2-a** under 590 nm light irradiation for 5 min; Figure S20: Fluorescence spectra of HPF in PBS dispersion of (a) 1-a and (b) 2-a under 590 nm light irradiation for 50 s; Figure S21: Relative absorption variation in (a) 1-Glc and (b) 2-Glc in PBS buffer exposed under 590 nm light irradiation for 50 min, (c) Corresponding relative absorbance variation at 520 nm of 1-Glc and 2-Glc in PBS buffer for 50 min; Figure S22: Ratio of quantitative fluorescence intensity in cells incubated with aggregates or glycol-dots of 1 and 2; Figure S23: CLSM images of calcein AM and propidium iodide PI co-staining HepG2 cells for live-dead cells after treating with 2-Glc and irradiating with or without white LED light; Figure S24: (a) The merge and brightfield images of DCFH-DA sensing the intracellular ROS generation after treating with 2-Glc and irradiating with or without white LED light, (b) Relative fluorescence intensity of DCFH-DA in each cell group after treating with 2-Glc and irradiating with or without white LED light; Figure S25: Statistical relative activity of *E. coli* incubated with different concentrations of 2-a and 2-Glc in illuminated conditions. Plate images of *E. coli* incubated with different concentrations of 2-a and 2-Glc in illuminated conditions; Figure S26: ^1^H-NMR of compound 1-S1 in CDCl_3_; Figure S27: ^1^H-NMR of compound 2-S1 in CDCl_3_; Figure S28: ^1^H-NMR and ^13^C-NMR of compound 1 in CDCl_3_; Figure S29: ^1^H-NMR and ^13^C-NMR of compound 2 in CDCl_3_; Figure S30: HRMS spectrum of 1; Figure S31: HRMS spectrum of 2.
