# Supplementary material for: Flexible Formation of Nanoparticles: Selectively Self-Assembling with Glycoclusters to Form Nano-Photosensitizers for Multipurpose Bioimaging and Photodynamic Therapy
Source: Molecules. 2025 Mar 12;30(6):1274. doi: 10.3390/molecules30061274 (PMC11944942; doi:10.3390/molecules30061274)
Supplement: Supplementary file 1 [file molecules-30-01274-s001.zip › molecules-3509079-supplementary.pdf]

Flexible Formation of Nanoparticles: Selectively Self-Assembling with Glycoclusters to Form Nano-Photosensitizers for Multipurpose Bioimaging and Photodynamic Therapy

Kai-Li He [1,†], Wen-Jia Li [2,3,4,†], Yu Hu [1], Lu-Lu Sun [2], Lei Dong [1,*], Jing Xing [1], Jin Gong [1,*], Xiaoming Gong [5,*] and Hai-Hao Han [2,3,4,*]

[1] School of Pharmacy, Shandong Second Medical University, Weifang 261053, China; hekaili2023@163.com (K.-L.H.); 13893624832@163.com (Y.H.); xjing1790@163.com (J.X.)
[2] Shandong Laboratory of Yantai Drug Discovery, Bohai Rim Advanced Research Institute for Drug Discovery, Yantai 264117, China; liwenjia@simm.ac.cn (W.-J.L.); llsun@baridd.ac.cn (L.-L.S.)
[3] Molecular Imaging Center, Stake Key Laboratory of Chemical Biology, Shanghai Institute of Materia Medica, Chinese Academy of Sciences, Shanghai 201203, China
[4] University of Chinese Academy of Sciences, Beijing 100049, China
[5] Comprehensive Technical Service Center of Weifang Customs, Weifang 261041, China
* Correspondence: leidong@sdsmu.edu.cn (L.D.); gongjin@sdsmu.edu.cn (J.G.); salmon_g@hotmail.com (X.G.); hanhaihao@simm.ac.cn (H.-H.H.)
† These authors contributed equally to this work.

Supporting Information

Flexible Formation of Nanoparticles: Selectively Self-assembling with Glycoclusters to Form Nano-photosensitizers for Multipurpose Bioimaging and Photodynamic Therapy

Kai-Li He,[a‡] Wen-Jia Li,[b,c,d‡] Yu Hu,[a] Lu-Lu Sun,[b] Lei Dong,[a]* Jing Xing,[a] Jin Gong, [a]* Xiaoming Gong,[e]* and Hai-Hao Han[b,c,d]*

[a] School of Pharmacy, Shandong Second Medical University, Weifang, Shandong 261053 China.

[b] Shandong Laboratory of Yantai Drug Discovery, Bohai Rim Advanced Research Institute for Drug Discovery, Yantai, Shandong 264117, China.

[c] Molecular Imaging Center, Stake Key Laboratory of Chemical Biology, Shanghai Institute of Materia Medica, Chinese Academy of Sciences, Shanghai 201203, China

[d] University of Chinese Academy of Sciences, Beijing 100049, P. R. China.

[e] Comprehensive Technical Service Center of Weifang Customs, Weifang, Shandong 261041, P. R. China

‡ Equal contribution

*Corresponding author: *leidong@sdsmu.edu.cn, hanhaihao@simm.ac.cn, gongjin@sdsmu.edu.cn, salmon_g@hotmail.com*

Contents List

Synthesis of photosensitizers **1** and **2**	S1-S3
Experimental section	S4-S6
Additional figures and schemes	S7-S21
References	S22

Synthesis of photosensitizers 1 and 2

General methods

All reagents for synthesis commercially available (highest purity available for reagent grade compounds) were used without further purification. Reactions under microwave activation were performed on a Biotage Initiator system. Thin-layer chromatography (TLC) was carried out on aluminum sheets coated with silica gel 60 F_{254} (Merck). TLC plates were inspected by UV light (λ = 254 nm, 365 nm) and developed by treatment with a mixture of 10% H_2SO_4 in EtOH/H_2O (95:5 v/v) followed by heating. Silica gel column chromatography was performed with silica gel Si 60 (40–63 µm). NMR spectra were recorded at 293 K, unless stated otherwise. Chemical shifts are referenced relative to deuterated solvent residual peaks. The following abbreviations are used to explain the observed multiplicities: s, singlet; d, doublet; t, triplet; q, quadruplet; m, multiplet; p, pseudo and b, broad.

Synthesis procedures

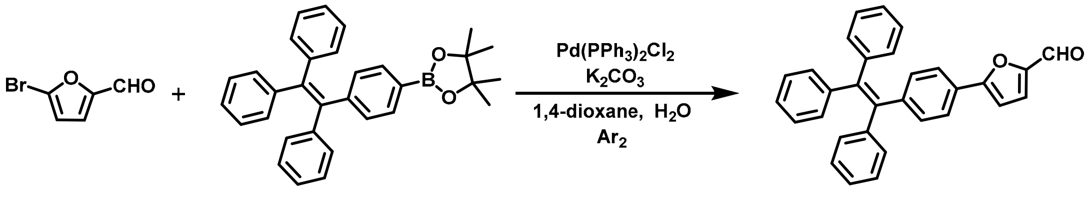

1-S1

*Synthesis procedure of compound **1-S1***:

To a solution of 5-bromofuran-2-carbaldehyde (500 mg, 2.86 mmol, 1 eq.) and 4,4,5,5-tetramethyl-2-(4-(1,2,2-triphenylvinyl)phenyl)-1,3,2-dioxaborolane (1.70 g, 3.71 mmol, 1.3 eq.) in 1,4-dioxane (15 mL) was added $Pd(PPh_3)_2Cl_2$ (61 mg, 0.085 mmol, 0.03 eq.) and potassium carbonate (1.18 g, 8.57 mmol, 3 eq.) and deionized water (5 mL). The resulting mixture was bubbled under Ar_2 during 10 min, and then heated at 100°C during 12 h under Ar_2. After cooling to room temperature (RT), the reaction was filtered through diatomite pad, diluted with EtOAc (50 mL), washed with brine (40 mL × 3). The combined organic layer was dried (Na_2SO_4), concentrated and purified with silica gel column chromatography (PE : EtOAc = 4:1) to afford compound **1-S1** (1.22 g, 61%) as a yellow powder.

^{1}H NMR (600 MHz, $CDCl_3$): δ (ppm) 9.61 (s, 1H), 7.56 (d, *J* = 8.4 Hz, 2H), 7.28 (d, *J* = 3.7 Hz, 1H), 7.14 – 7.08 (m, 12H), 7.07 – 7.00 (m, 5H), 6.75 (d, *J* = 3.8 Hz, 1H).

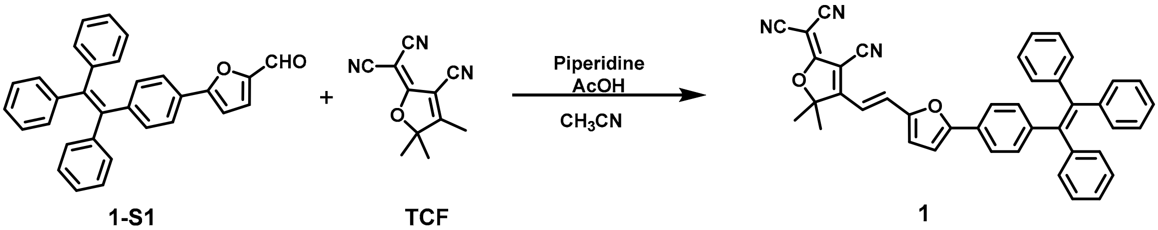

1-S1 **TCF** **1**

*Synthesis procedure of compound **1***:

To a solution of **1-S1** (245 mg, 0.574 mmol, 1 eq.) and 2-(3-cyano-4,5,5-trimethylfuran-2(*5H*)-ylidene)malononitrile (TCF, 137 mg, 0.689 mmol, 1.2 eq.) in CH_3CN (20 mL) was added piperidine (0.3 mL) and AcOH (0.3 mL). The mixture was refluxed until TCL monitored the disappearance of starting materials. The reaction was diluted with EtOAc (50 mL), washed with HCl aqueous solution (2M, 40 mL) and brine (40 mL × 3). The combined organic layer was dried (Na_2SO_4), concentrated and

purified with silica gel column chromatography (PE : EtOAc = 3:1) to afford compound **1** (231 mg, 66%) as a dark purple powder.

^{1}H NMR (600 MHz, $CDCl_3$): δ (ppm) 7.54 (d, *J* = 8.5 Hz, 2H), 7.50 (d, *J* = 15.8 Hz, 1H), 7.17 – 7.09 (m, 12H), 7.09 – 7.06 (m, 2H), 7.05 – 7.00 (m, 4H), 6.83 (d, *J* = 3.8 Hz, 1H), 6.78 (d, *J* = 15.8 Hz, 1H), 1.74 (s, 6H).

^{13}C NMR (151 MHz, $CDCl_3$): δ (ppm) 176.0, 173.3, 161.5, 160.8, 160.6, 150.7, 146.4, 143.7, 143.6, 143.5, 142.7, 140.3, 132.5, 131.71, 131.69, 131.6, 128.4, 128.3, 128.2, 128.1, 127.2, 127.1, 124.9, 112.4, 111.6, 111.1, 110.4, 97.5, 57.0, 26.7.

HR-ESI-MS *m/z*: calcd. for $C_{42}H_{29}N_3O_2Na^+$ $[M+Na]^+$ 630.2152, found 630.2142.

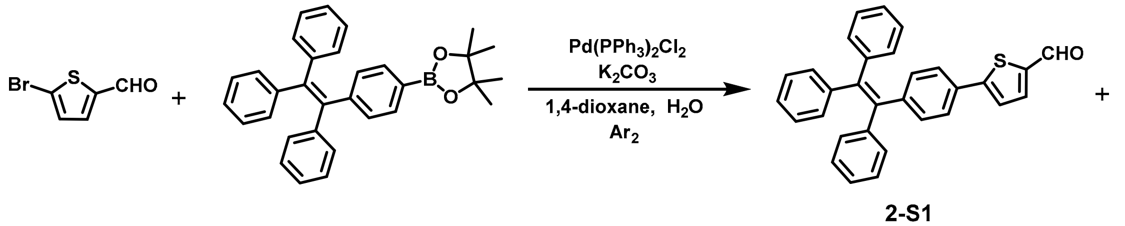

2-S1

*Synthesis procedure of compound **2-S1***:

To a solution of 5-bromothiophene-2-carbaldehyde (500 mg, 2.62 mmol, 1 eq.) and 4,4,5,5-tetramethyl-2-(4-(1,2,2-triphenylvinyl)phenyl)-1,3,2-dioxaborolane (1.56 g, 3.40 mmol, 1.3 eq.) in 1,4-dioxane (15 mL) was added $Pd(PPh_3)_2Cl_2$ (55 mg, 0.079 mmol, 0.03 eq.) and potassium carbonate (1.09 g, 7.85 mmol, 3 eq.) and deionized water (5 mL). The resulting mixture was bubbled under Ar_2 during 10 min, and then heated at 100°C during 12 h under Ar_2. After cooling to room temperature (RT), the reaction was filtered through diatomite pad, diluted with EtOAc (50 mL), washed with brine (40 mL × 3). The combined organic layer was dried (Na_2SO_4), concentrated and purified with silica gel column chromatography (PE : EtOAc = 4:1) to afford compound **2-S1** (725 mg, 63%) as a yellow powder.

^{1}H NMR (600 MHz, $CDCl_3$): *δ* (ppm) 9.86 (s, 1H), 7.70 (d, *J* = 3.9 Hz, 1H), 7.42 (d, *J* = 8.4 Hz, 2H), 7.33 (d, *J* = 3.9 Hz, 1H), 7.15 – 7.07 (m, 13H), 7.07 – 7.01 (m, 4H).

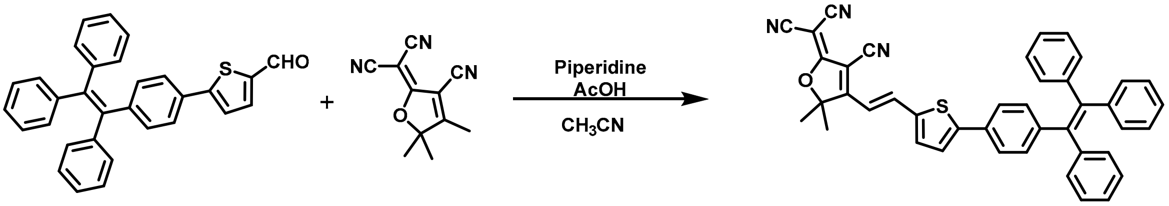

2-S1 **TCF** **2**

*Synthesis procedure of compound **2***:

To a solution of **2-S1** (300 mg, 0.677 mmol, 1 eq.) and TCF (162 mg, 0.813 mmol, 1.2 eq.) in CH_3CN (20 mL) was added piperidine (0.3 mL) and AcOH (0.3 mL). The mixture was refluxed until TCL monitored the disappearance of starting materials. The reaction was diluted with EtOAc (50 mL), washed with HCl aqueous solution (2M, 40 mL) and brine (40 mL × 3). The combined organic layer was dried (Na_2SO_4), concentrated and purified with silica gel column chromatography (PE : EtOAc = 3:1) to afford compound **2** (228 mg, 54%) as a dark purple powder.

^{1}H NMR (600 MHz, DMSO-*d*$_6$): *δ* (ppm) 7.80 (d, *J* = 15.8 Hz, 1H), 7.43 (d, *J* = 4.0 Hz, 1H), 7.40 (d, *J* = 8.2 Hz, 2H), 7.33 (d, *J* = 4.1 Hz, 1H), 7.15 – 7.13 (m, 6H), 7.12 – 7.10 (m, 4H), 7.10 – 7.05 (m, 3H), 7.06 – 7.00 (m, 4H), 6.65 (d, *J* = 15.9 Hz, 1H), 1.76 (s, 6H).

^{13}C NMR (150 MHz, DMSO-*d*$_6$): *δ* (ppm) 175.8, 173.4, 153.5, 146.1, 143.7, 143.6, 143.5, 142.7, 140.2, 139.8, 139.1, 137.1, 132.6, 131.69, 131.66, 131.6, 128.3, 128.2, 128.0, 127.2, 127.12, 127.08, 125.8, 125.5, 113.0, 112.2, 111.5,

111.0, 97.5, 57.3, 26.8.

HR-ESI-MS *m/z*: calcd. for $C_{42}H_{29}N_3OSNa^+$ $[M+Na]^+$ 646.1924, found 646.1918.

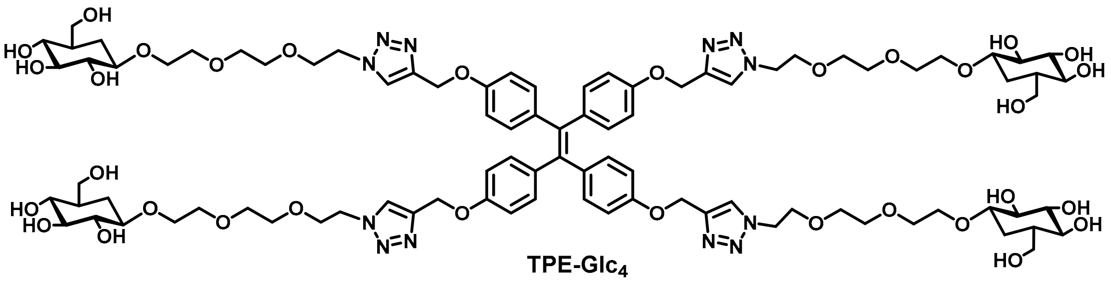

*Synthesis procedure of compound **TPE-Glc$_4$***:

The synthesis processes of **TPE-Glc$_4$** have been reported in our previous works.[2]

Experimental section

Material and instruments. Materials were obtained from commercial suppliers and were used without further purification. All reactions were performed in oven-dried glassware (Hinwil) unless otherwise stated. Column chromatography was performed over silica gel (200-300 mesh). NMR spectra were recorded with JEOL-400 or JEOL-600 spectrometers. High-resolution mass spectrometry experiments were recorded by Bruker Solarix XR Fourier Transform Ion Cyclotron Resonance Mass Spectrometer. All optical spectra were recorded at room temperature. Absorption spectra of liquid samples were determined on Hitachi UV-3900 spectrophotometer. Fluorescence spectra of liquid samples were determined on determined on Hitachi F-4600 spectrophotometer. The absolute quantum yield was determined on Hamamatsu Quantaurus-QY. Dynamic light scattering (DLS) investigations were carried out with a DynaPro NanoStar dynamic light scattering detector. Scanning electron microscope (SEM) images were obtained using a Hitachi SU-8010 instrument. The photostability was conducted under irradiation with a high-power LED light and monitored by using a UV-3900 spectrophotometer. The cell fluorescence imaging experiments were conducted with a laser scanning confocal microscopy (Olympus-FV3000, Olympus, Tokyo, Japan) Cell viability was measured on an EnVision multilabel plate reader (PerkinElmer, USA). Irradiation was performed by using a LED light (590 nm, PLS-LED 100, Perfect Light, Beijing, China).

UV-Vis absorption. The UV-Vis absorption spectra were measured at room temperature using a Hitachi UV-3900 spectrophotometer. All spectra were corrected for background intensities by subtracting the spectra of pure solvent measured under identical conditions. The absorbance of **1** and **2** (c = 5 μM) in monomer state were measured in various organic solvents. The relative absorption (*Abs*) was normalized the absorbance into [0,1] based on the maxima in figures.

Fluorescence spectroscopy. The fluorescence measurements were carried out at room temperature using Hitachi F-4600 spectrophotometer. The fluorescence emission of **1** and **2** (c = 5 μM) in monomer state was measured in various organic solvents (λ_{ex} = 520 nm, slit width 5-10 nm, 700 V). d. The relative emission intensity (I_F) was normalized the emission intensity into [0,1] based on the maxima in figures.

Absolute fluorescence quantum yield. 1 or **2** was diluted in 1,4-dioxane with concentration of 5 μM. The absolute fluorescence quantum yield was determined by Hamamatsu Quantaurus-QY.

Theoretical calculations. Gaussian 16 program was used to perform density functional theory (DFT) calculations (b3lyp/6-31g(d,p)) of **1** and **2**.[3-6] The high-performance computing server was provided by Beijing Super Cloud Computing Center, Beijing, China.

Formation of amorphous aggregates. Compound **1** or **2** (1 mmol) was well-dissolved in DMSO (20 μL). The resulting solution was added 9980 μL deionized (DI) water or PBS buffer (pH 7.4, 1 mM) and then dispersed under ultrasound in ice bath for 10 min to form the amorphous aggregates (**1-a** or **2-a**, 100 μM). The resulting dispersion in water or PBS buffer was diluted to low concentration for spectral measurements.

Formation of glyco-dots. Compound **1** or **2** (1 mmol) was well-dissolved in DMSO (20 μL). **TPE-Glc$_4$** (10 mmol) was well-diluted in H_2O or PBS buffer (80 μL). The DMSO solution of **1** or **2** was mixed with **TPE-Glc$_4$**, and then added 9900 μL deionized (DI) water or PBS buffer. The resulting solution was sufficiently dispersed under ultrasound in

ice bath for 10 min to form the glycol-dots (**1-Glc** or **2-Glc**, 100 μM). The glyco-dots dispersion in water or PBS buffer was diluted to low concentration for spectral measurements.

Dynamic light scattering. The dispersion of amorphous aggregates (**1-a** or **2-a**) and glycol-dots (**1-Glc** or **2-Glc)** in H_2O was diluted to 50 μM, and then measured the hydrated size by DynaPro NanoStar dynamic light scattering detector.

Detection of ROS production by DCFH in solution. Commercial probe 2',7'-dichlorofluorescein (DCFH) was used as ROS detector. 10 μM of determinand were dissolved in 2 mL DMF containing 20 μM of DCFH. 10 μM of determinand (aggregates or glyco-dots) were prepared in 2 mL PBS buffer (pH 7.4, 1 mM) containing 20 μM of DCFH. The dispersion was then placed in a cuvette and irradiated with a 590 nm LED light at 10 mW cm^{-2} for 50 s. The fluorescence change of sample at 525 nm was recorded under excitation at 470 nm.

Detection of $^1O_2$ production in solution. Compound 9,10-anthracenediyl-bis(methylene)-dimalonic acid (ABDA) was used as indicator for detection of $^1O_2$ in solution. When $^1O_2$ is generated in the system, the ABDA will be oxidized, and the absorption decreases at 380 nm. 10 μM of determinand (aggregates or glyco-dots) were prepared in 2 mL PBS buffer (pH 7.4, 1 mM) containing 50 μM of ABDA. The dispersion was then placed in a cuvette and irradiated with a 590 nm LED light at 10 mW cm^{-2} for 150 s. The absorption decrease of sample at 380 nm was recorded by the UV-Vis absorption spectrophotometer.

Detection of $O_2^{-\bullet}$ production with DHR123 in solution. Commercial probe dihydrorhodamine 123 (DHR123) was used as $O_2^{-\bullet}$ detector. When $O_2^{-\bullet}$ is generated in the system, DHR123 can be oxidized to emit bright fluorescence at *ca.* 530 nm. 10 μM of determinand (aggregates or glyco-dots) were prepared in 2 mL PBS buffer (pH 7.4, 1 mM) containing 10 μM of DHR123. The mixture was then placed in a cuvette and irradiated with a 590 nm LED light at 10 mW cm^{-2} for 75 s. The fluorescence change of sample at 530 nm was recorded under excitation at 470 nm.

Detection of $O_2^{-\bullet}$ production with DHE in solution. Commercial probe dihydroethidium (DHE) was used as indicator for detection of $O_2^{-\bullet}$ in solution. When $O_2^{-\bullet}$ is generated in the system, DHE can be oxidized to form ethidium which intercalates into DNA and emits bright fluorescence at *ca.* 580 nm. 10 μM of determinand (aggregates or glyco-dots) were prepared in 2 mL PBS buffer (pH 7.4, 1 mM) containing 30 μM of DHE and 200 μg/mL ctDNA. The mixture was then placed in a cuvette and irradiated with a 590 nm LED light at 10 mW cm^{-2} for 5 min. The fluorescence change of sample at 600 nm was recorded under excitation at 470 nm.

Detection of ·OH production with HPF in solution. Commercial probe hydroxyphenyl fluorescein (HPF) was used as ·OH detector. When ·OH is generated in the system, HPF can be oxidized to emit bright fluorescence at *ca.* 520 nm. 10 μM of determinand (aggregates or glyco-dots) were prepared in 2 mL PBS buffer (pH 7.4, 1 mM) containing 10 μM of HPF. The mixture was then placed in a cuvette and irradiated with a 590 nm LED light at 10 mW cm^{-2} for 50 s. The fluorescence change of sample at 520 nm was recorded under excitation at 470 nm.

Nanoparticle stability at room temperature. The prefabricated glycol-dots of **1-Glc** or **2-Glc** (200 μL, 100 μM) were diluted in PBS buffer (pH 7.4, 1800 μL) to afford the diluted concentration (10 μM), and placed at room temperature. Absorption spectra were determined within 6 days (interval 1 d).

Nanoparticle photostability. The prefabricated glycol-dots of **1-Glc** or **2-Glc** (200 μL, 100 μM) were diluted in PBS

buffer (pH 7.4, 1800 μL) to afford the diluted concentration (10 μM). The resulting diluted nanoparticle dispersion in PBS buffer was exposed under 590 nm LED light irradiation (P = 30 mW cm^{-2}) during 50 min. Absorption spectra were determined within 50 min (interval 10 min).

***In vitro* dark / light cytotoxicity.** HepG2 cells were seeded in 96-well plates (8×10^3 cells/well) and incubated for 24 h under normoxia. Then, the medium was replaced with 100 μL of DMEM supplemented with 1% DMSO containing different concentrations (0-20 μM) of glycol-dots. After incubation for another 4 h, the cells were washed three times with PBS, infused with fresh medium, and illuminated by a white LED light (30 mW cm^{-2}) for 2 h. After that, the cell viability was examined by cell counting kit-8 (CCK-8) assays. All experiments were repeated three times with representative data shown.Moreover, the dark toxicity of glycol-dots was also analysed by the above procedure except the illumination was eliminated.

Cell uptake and imaging. HepG2 cells were seeded on 24-well plates (3×10^4 cells/well) cultured in DMEM medium containing 10% FBS under normoxia for 24 h. Then, the medium was replaced with culture medium with aggregates (**1-a** or **2-a**, 10 μM) or glycol-dots (**1-Glc** or **2-Glc**, 10 μM) and then further incubated for 2 h. Subsequently, the cells were washed three times with PBS and imaged by confocal microscopy.

Calcein-AM/PI co-staining of HepG2 cells in PDT experiments. The HepG2 cells were seeded on 24-well plates (8×10^4 cells/well) for 24 h and then treated by **2-Glc** (20 μM) and irradiation under white LED light (30 mW cm^{-2}) for 2 h. The treated cells were stained with PBS buffer mixed Calcein-AM (2 μM) and PI (1 μg/mL) for 30 min in the cell-cultured incubator. After PBS wash, the cell samples were imaged on confocal microscopy with the excitation at 488 nm (emission 510-560 nm) and 561 nm (emission 620-670 nm), respectively.

Intracellular ROS generation. The DCFH-DA as an indicator was used to measure the production of ROS in cells. After culturing HepG2 cells with samples containing 20 μM **2-Glc** for 4 h, the medium was replaced with medium containing DCFH-DA (5 μM) and incubated for 30 min. After exposure to white light (30 mW cm^{-2}) for 2 h, the cells were washed with PBS three times and imaged by confocal microscopy.

Bacterial toxicity assay. An equal volume of bacterial solution (100 μL, *ca.* 1×10^5 CFU mL^{-1}) was added to the diluted photosensitizer solution in a 48 well plate and the 48 well plate was incubated at 37°C for 1 h. The dispersions of **2-a** and **2-Glc** were added to the bacterial solution with different final concentrations as 0, 4, 8, 12, 16 μM and incubated for 80 min. After irradiating under white LED light for 30 min (30 mW cm^{-2}), 100 μL bacterial solution was coated on solid LB medium plate. After incubation at 37°C for 10 h, the number of bacterial single colonies on the solid LB medium plate was recorded. Minimum inhibitory concentration 90 (MIC_{90}) was determined as the lowest concentration of PSs at which we observed bacterial relative activity was inhibited to >90%. The dark toxicity assay of amorphous aggregates and glycol-nanoparticles were analyzed at same experimental conditions but without the light irradiation.

Additional figures and schemes

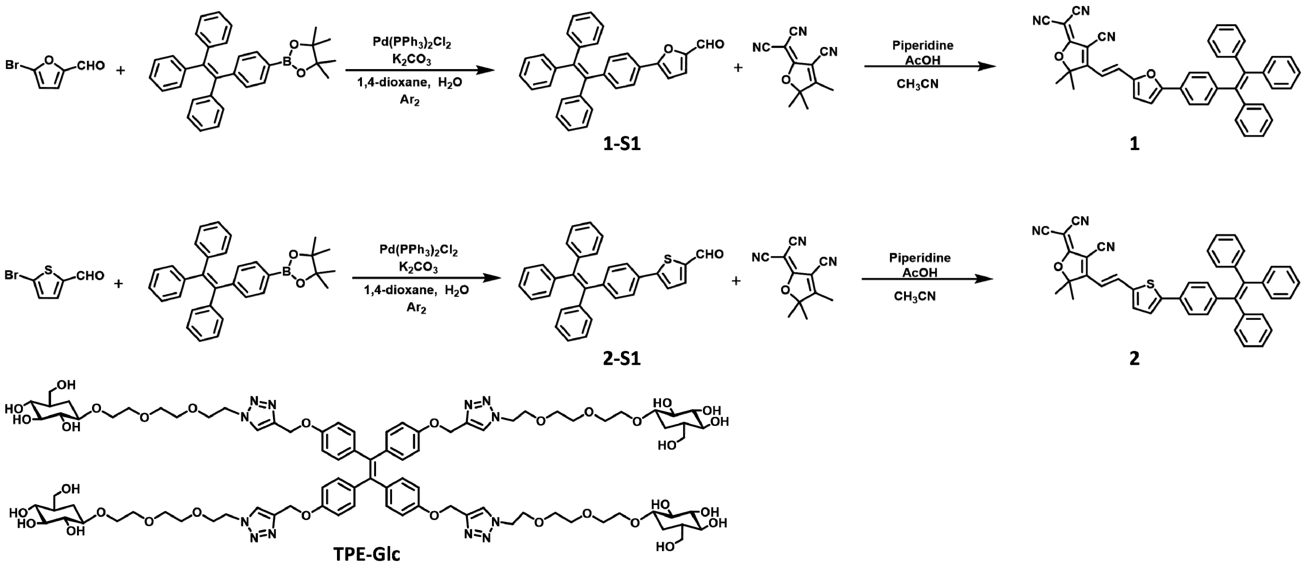

Scheme S1 Synthesis procedures of photosensitizers **1** and **2**.

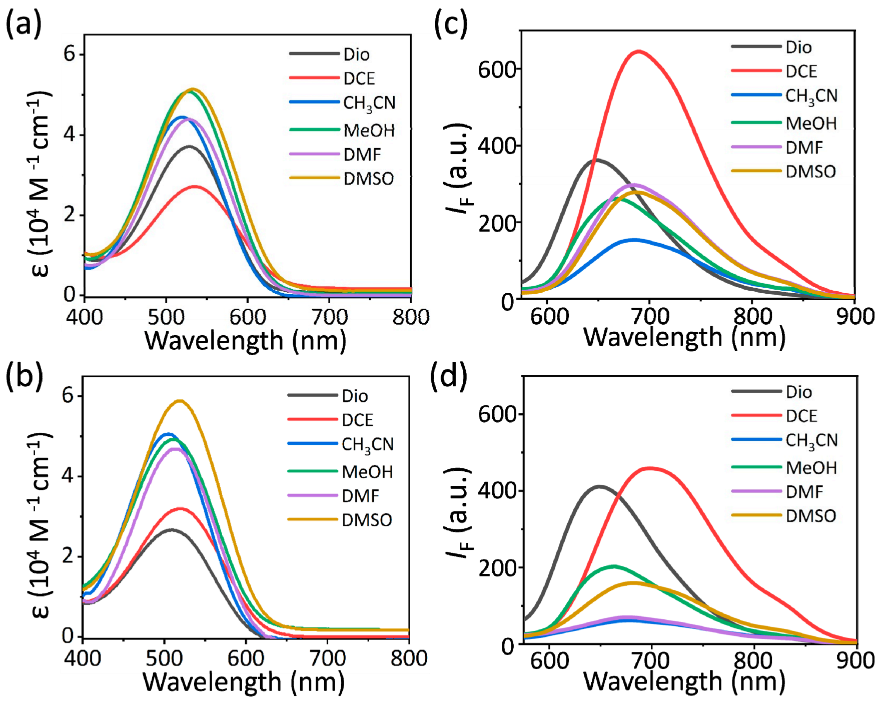

Figure S1. Absorption spectra and molar extinction coeffcient (*ε*) of (a) **1** and (b) **2** (c = 5 μM) in different organic solvents. Fluorescence spectra of (c) **1** and (d) **2** (c = 5 μM) in different organic solvents (1,4-dioxane (Dio), 1,2-dichloroethane (DCE), CH_3CN, MeOH, DMF and DMSO).

Table S1. Photophysical properties of **1** and **2** in various solvents

	Solvent	λ_{abs} (nm) [1]	ε (M^{-1} cm^{-1})	λ_{em} (nm) [2]	Stokes Shift (nm)
	1,4-dioxane	526	30400	648	122
	1,2-dichloroethane	534	23400	689	155
	CH_3CN	518	36800	684	166
**1**	MeOH	530	42000	669	139
	DMF	530	36400	687	157
	DMSO[3]	534	42400	689	155
	H_2O	525	21200	716	191
	1,4-dioxane	508	26700	650	142
	CH_2Cl_2	520	31900	699	179
	CH_3CN	504	50500	680	176
**2**	MeOH	512	49100	663	151
	DMF	513	46800	676	163
	DMSO[4]	520	58700	685	165
	H_2O	510	20900	720	210

[1] Wavelength of absorption maxima. [2] Wavelength of fluorescence maxima. [3] Absolute fluorescence quantum yield of **1** in DMSO is 1.9%. [4] Absolute fluorescence quantum yield of **2** in DMSO is 1.6%.

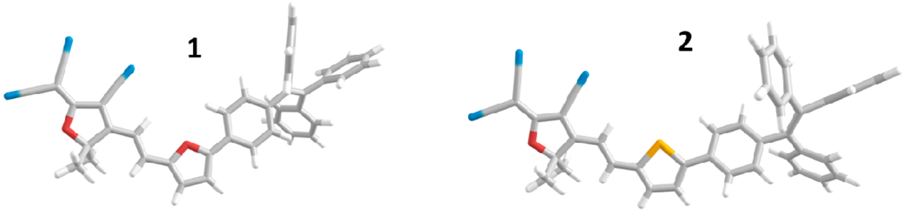

Figure S2. Density functional theory (DFT) calculated the molecular optimal conformation of **1** and **2**.

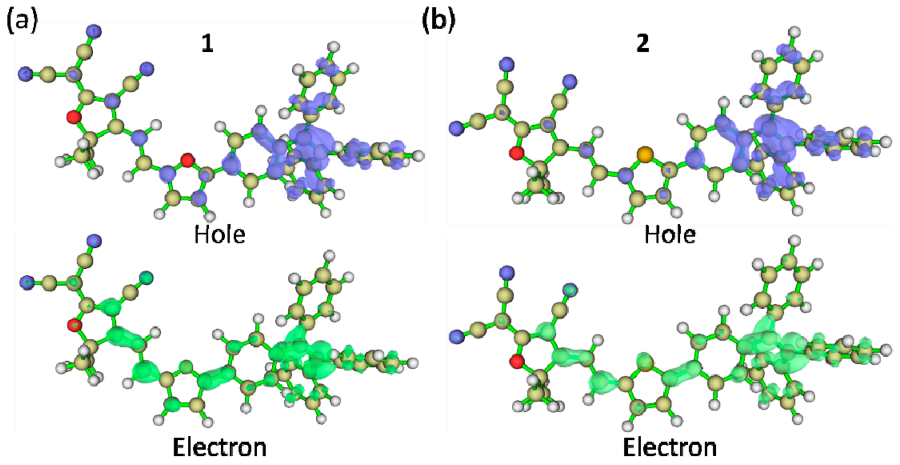

Figure S3. DFT calculated the electron-hole distribution of (a) **1** and (b) **2** at singlet excited (S_1) state.

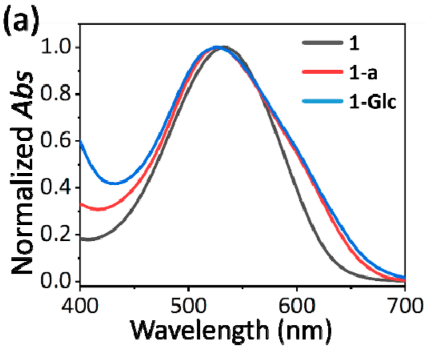

Figure S4. Normalized absorption of compounds (a) **1** and (b) **2** within monomer (grey), amorphous aggregates (red) and glycol-dots (blue) states (c = 5 μM).

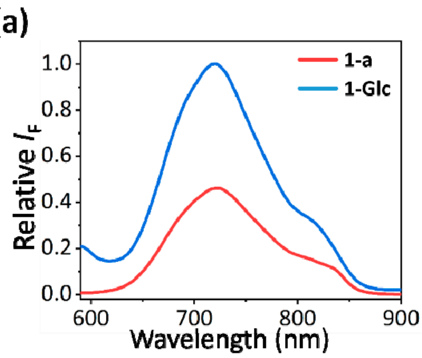

Figure S5. Relative fluorescence spectra of compound (a) **1** and (b) **2** within amorphous aggregates (red) and glycol-dots (blue) states (c = 5 μM, λ_{ex} = 520 nm).

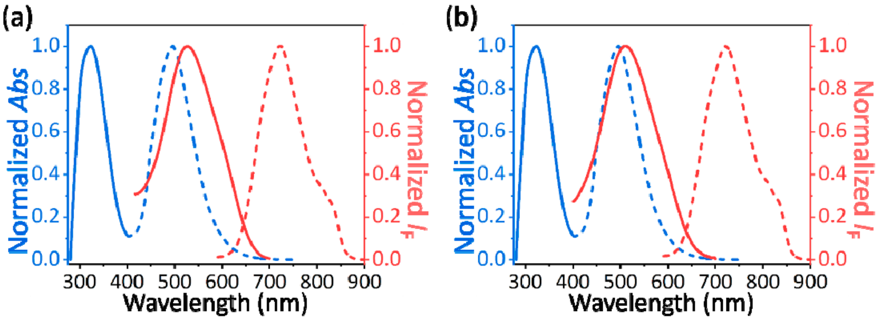

Figure S6. Normalized absorption (line) and fluorescence (dash line) spectra of **TPE-Glc$_4$** (blue line) compared with that of (a) **1** and (b) **2** (red line).

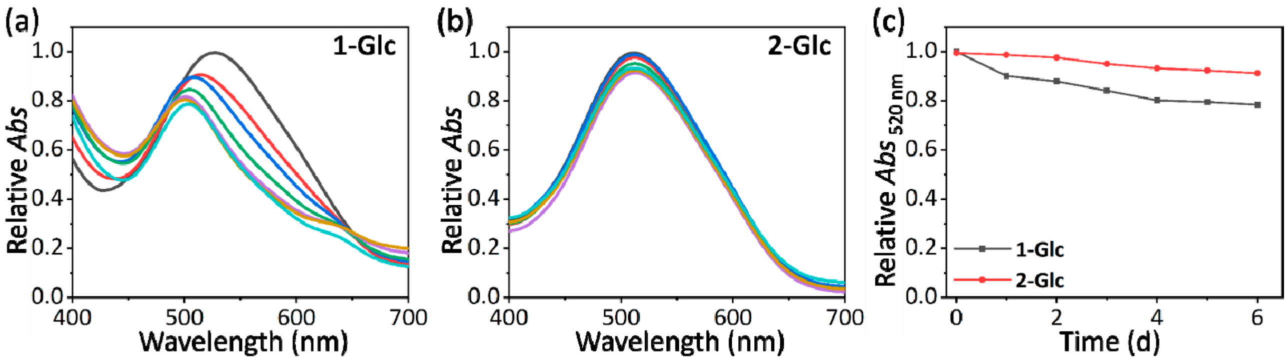

Figure S7. Relative absorption spectra of (a) **1-Glc** and (b) **2-Glc** in PBS buffer at room temperaute for 6 d (interval 1 day, c = 10 μM). (c) Corresponding relative absorbance variation at 520 nm of **1-Glc** and **2-Glc** in PBS buffer for 7 d.

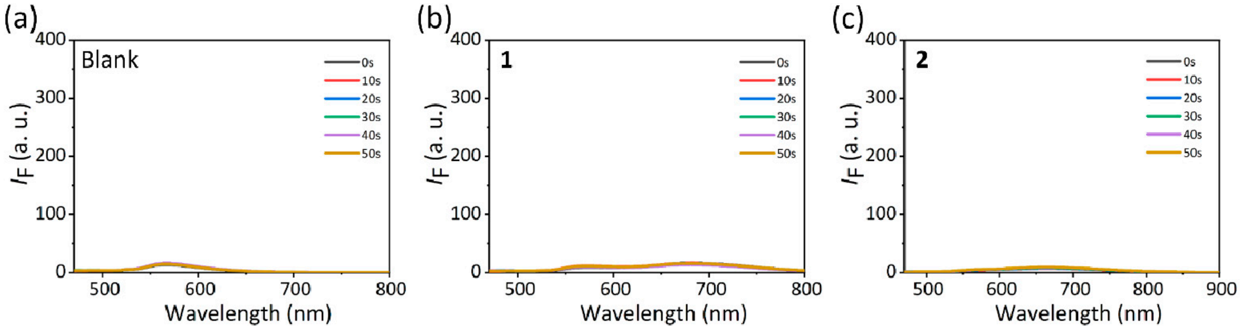

Figure S8. Fluorescence spectra of DCFH in DMF solution of (a) blank, (b) **1** and (c) **2** (c = 10 μM) under 590 nm light irradiation for 50 s (interval 10 s, P = 10 mW cm^{-2}, λ_{ex} (DCFH) = 470 nm).

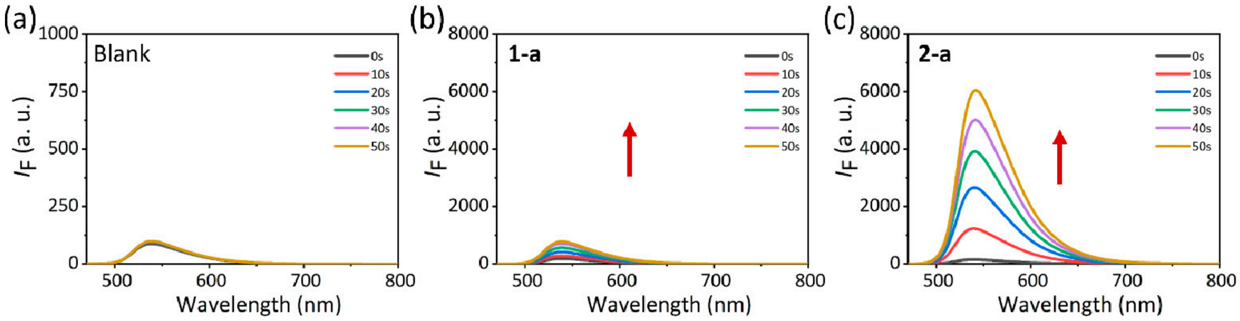

Figure S9. Fluorescence spectra of DCFH in PBS of (a) blank, (b) **1-a** and (b) **2-a** (c = 10 μM) under 590 nm light irradiation for 50 s (interval 10 s, P = 10 mW cm^{-2}, λ_{ex} (DCFH) = 470 nm).

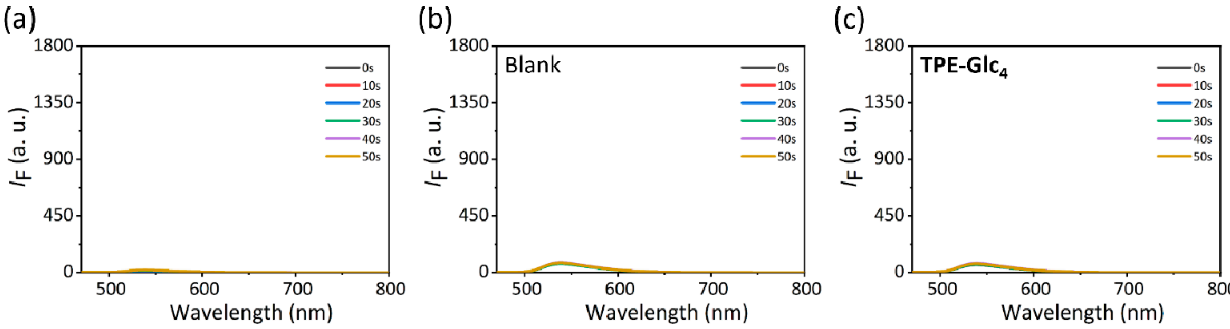

Figure S10. (a) Fluorescence spectra of **TPE-Glc$_4$** (c = 50 μM) in PBS under 590 light irradiation for 50 s (interval 10 s, λ_{ex} (**TPE-Glc$_4$**) = 470 nm, P = 10 mW cm^{-2}). (b) Fluorescence spectra of DCFH in PBS buffer without PSs (blank sample) under 590 nm light irradiation for 50 s. (c) Fluorescence spectra of DCFH in PBS dispersion of **TPE-Glc$_4$** (c = 50 μM) under 590 nm light irradiation for 50 s (interval 10 s, P = 10 mW cm^{-2}, λ_{ex} (DCFH) = 470 nm).

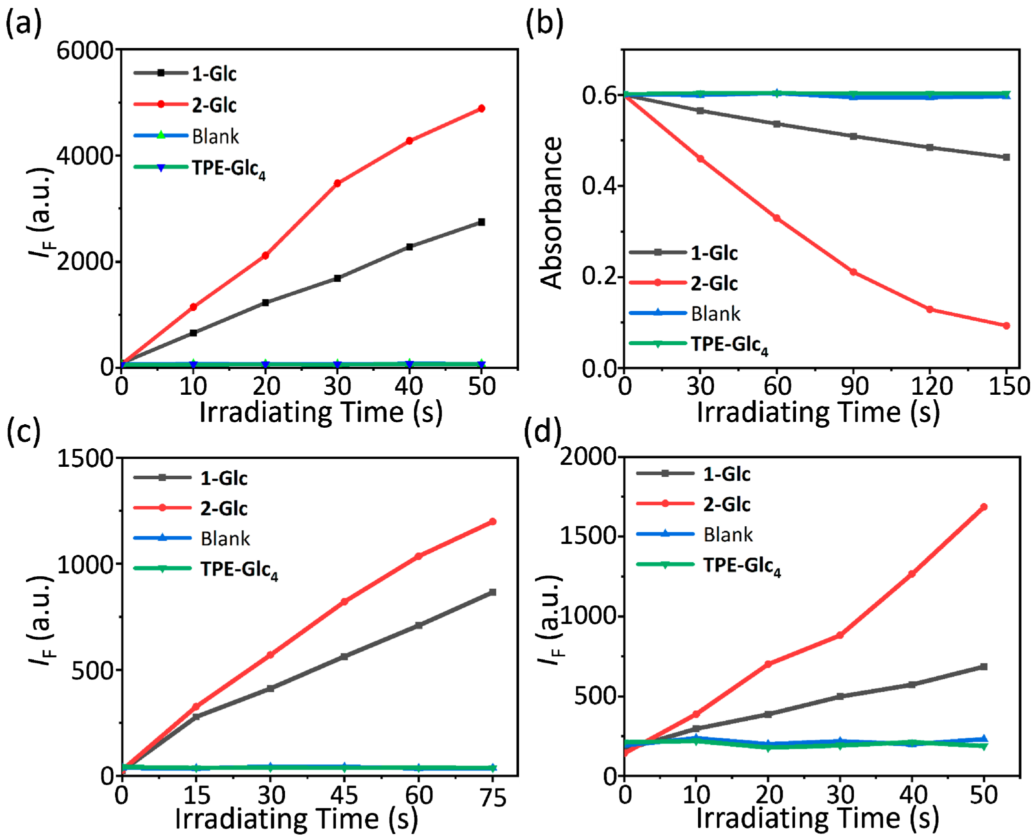

Figure S11. (a) Fluorescence variation of DCFH in PBS dispersion of **1-Glc** and **2-Glc** (c = 10 μM) under light for 50 s (interval 10 s, λ_{ex} (DCFH) = 470 nm). (b) Absorption variation of ABDA in PBS **1-Glc** and **2-Glc** (c = 10 μM) under light for 150 s (interval 30 s, P = 10 mW cm^{-2}). (c) Fluorescence variation of DHR123 in PBS dispersion of **1-Glc** and **2-Glc** (c = 10 μM) under light for 75 s (interval 15 s, λ_{ex} (DHR123) = 470 nm). (d) Fluorescence variation of HPF in PBS dispersion of **1-Glc** and **2-Glc** (c = 10 μM) under light for 50 s (interval 10 s, λ_{ex} (HPF) = 470 nm, 590 nm LED light, P = 10 mW cm^{-2})

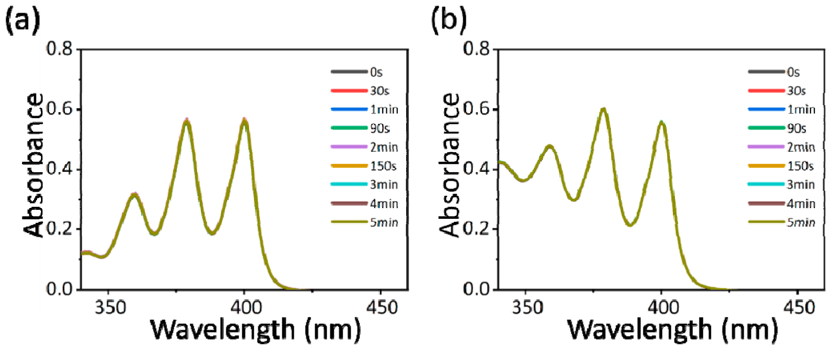

Figure S12. (a) Absorption spectra of ABDA in PBS buffer without PSs (blank sample) under 590 nm light irradiation for 5 min. (b) Absorption spectra of ABDA in PBS dispersion of **TPE-Glc$_4$** (c = 50 µM) under 590 nm light irradiation for 5 min (P = 10 mW cm^{-2}).

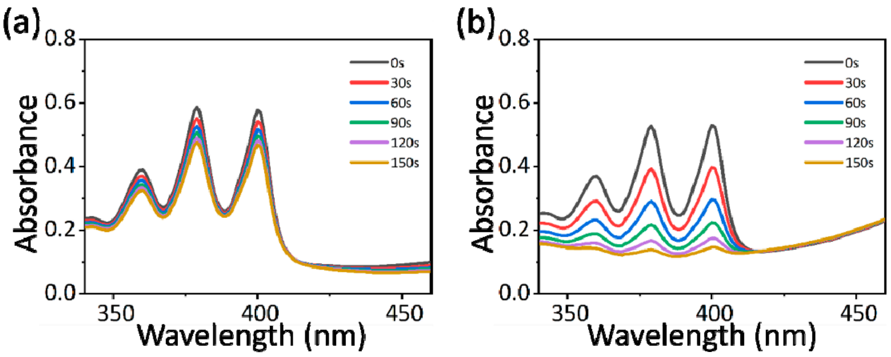

Figure S13. Absorption spectra of ABDA in PBS dispersion of (a) **1-a** and (b) **2-a** (c = 10 µM) under 590 nm light irradiation for 150 s (interval 30 s, P = 10 mW cm^{-2}).

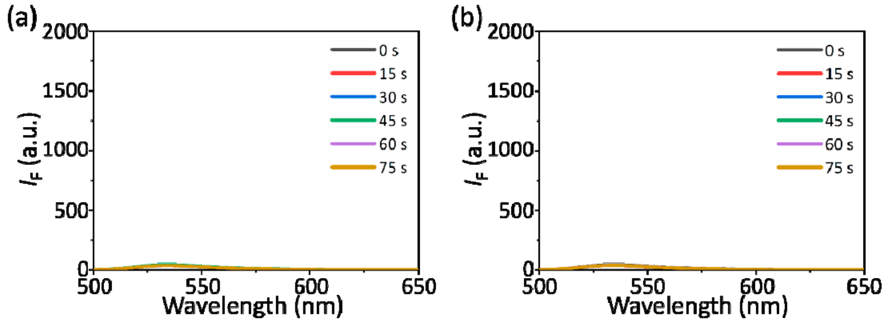

Figure S14. (a) Fluorescence spectra of DHR123 in PBS buffer without PSs (blank sample) under 590 nm light irradiation for 75 s. (b) Fluorescence spectra of DHR123 in PBS dispersion of **TPE-Glc$_4$** (c = 50 µM) under 590 nm light irradiation for 75 s (interval 15 s, P = 10 mW cm^{-2}, λ_{ex} (DHR123) = 470 nm).

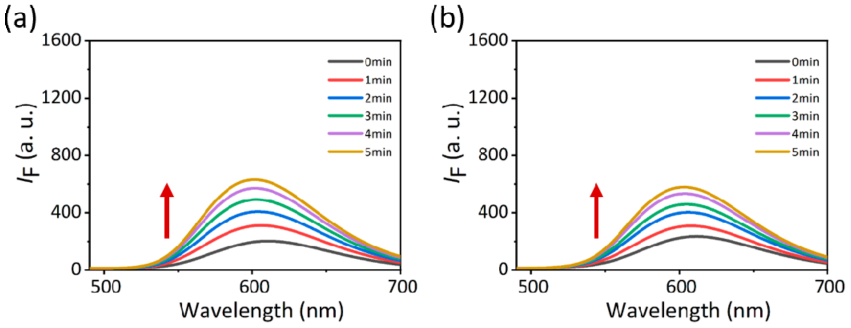

Figure S15. (a) Fluorescence spectra of DHE mixed with DNA in PBS buffer without PSs (blank sample) under 590 nm light irradiation for 5 min. (b) Fluorescence spectra of DHE mixed with DNA in PBS dispersion of **TPE-Glc$_4$** (c = 50 μM) under 590 nm light irradiation for 5 min (interval 1min, λ_{ex} (DHE) = 470 nm, P = 10 mW cm^{-2}).

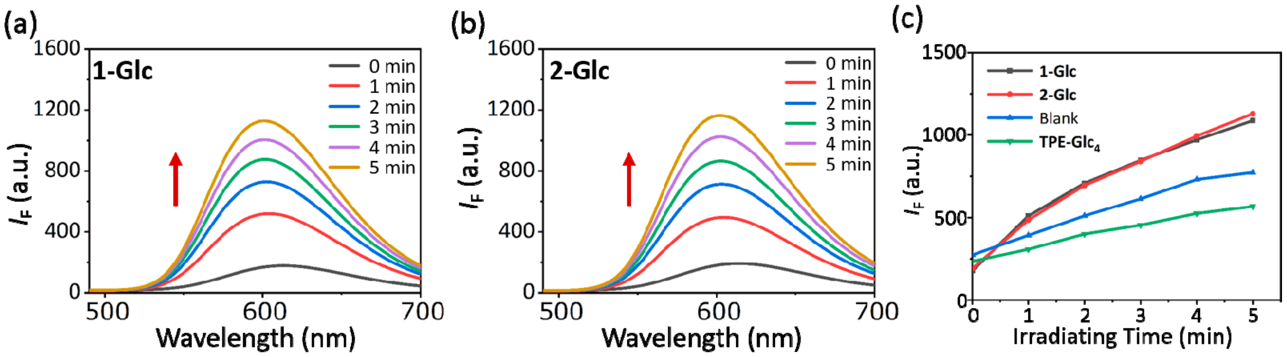

Figure S16. Fluorescence spectra of DHE mixed with DNA in PBS dispersion of (a) **1-Glc** and (b) **2-Glc** (c = 10 μM) under light for 5 min (interval 1 min, λ_{ex} (DHE) = 470 nm). (c) Fluorescence variation of DHE mixed with DNA in PBS dispersion of **1-Glc** and **2-Glc** (c = 10 μM) under light for 5 min (interval 1min, λ_{ex} (DHE) = 470 nm, P = 10 mW cm^{-2}).

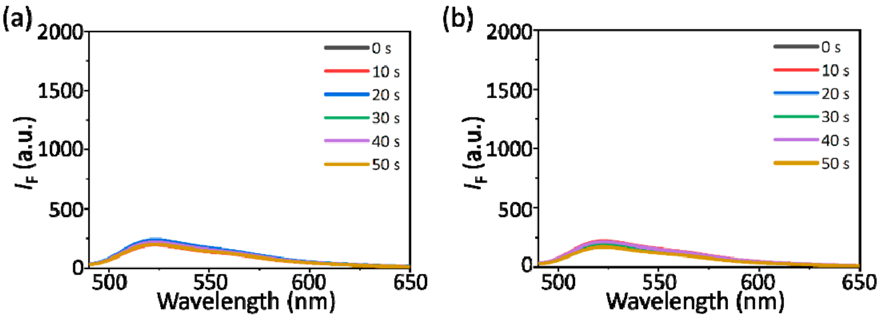

Figure S17. (a) Fluorescence spectra of HPF in PBS buffer without PSs (blank sample) under 590 nm light irradiation for 50 s. (b) Fluorescence spectra of HPF in PBS dispersion of **TPE-Glc$_4$** (c = 50 μM) under 590 nm light irradiation for 50 s (interval 10 s, P = 10 mW cm^{-2}, λ_{ex} (HPF) = 470 nm).

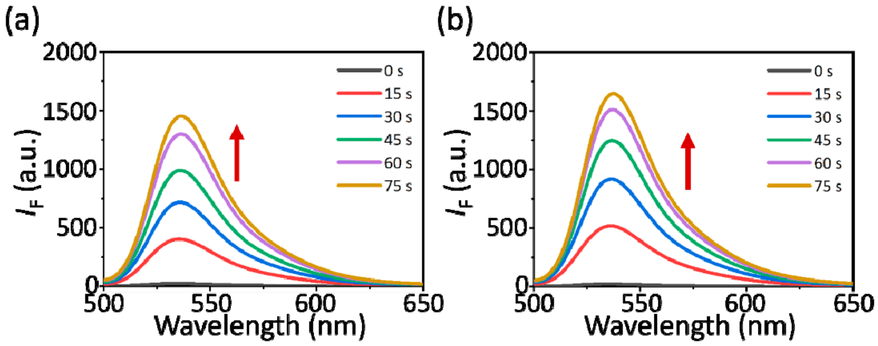

Figure S18. Fluorescence spectra of DHR123 in PBS dispersion of (a) **1-a** and (b) **2-a** (c = 10 μM) under 590 nm light irradiation for 75 s (interval 15 s, *P* = 10 mW cm^{-2}, λ_{ex} (DHR123) = 470 nm).

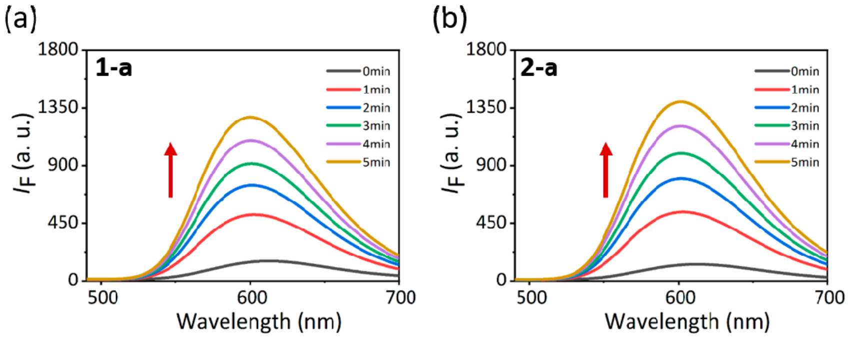

Figure S19. Fluorescence spectra of DHE mixed with DNA in PBS dispersion of (a) **1-a** and (b) **2-a** (c = 10 μM) under 590 nm light irradiation for 5 min (interval 1 min, *P* = 10 mW cm^{-2}, λ_{ex} (DHE) = 470 nm).

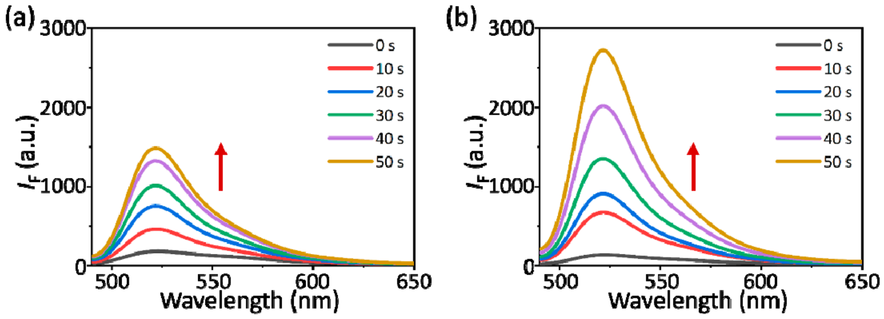

Figure S20. Fluorescence spectra of HPF in PBS dispersion of (a) **1-a** and (b) **2-a** (c = 10 μM) under 590 nm light irradiation for 50 s (interval 10 s, *P* = 10 mW cm^{-2}, λ_{ex} (HPF) = 470 nm).

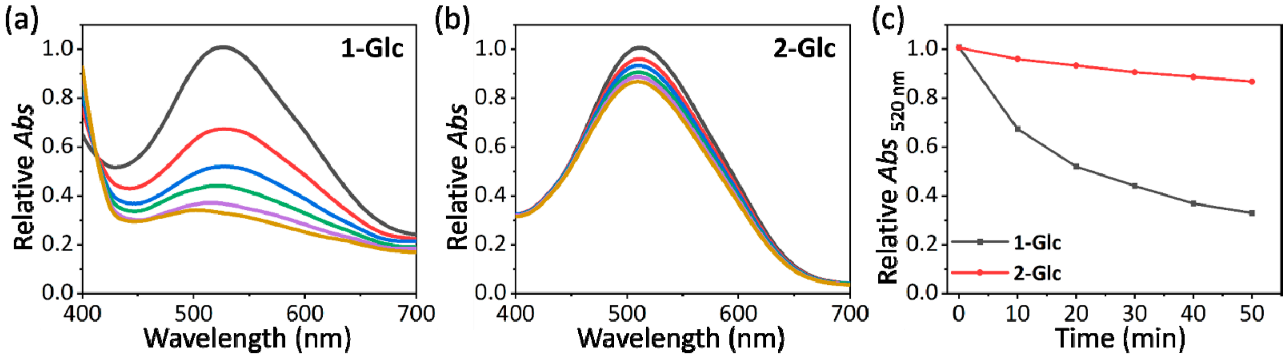

Figure S21. Relative absorption variation of (a) **1-Glc** and (b) **2-Glc** in PBS buffer exposed under 590 nm light irradiation for 50 min (interval 10 min, c = 10 μM, P = 30 mW cm^{-2}). (c) Corresponding relative absorbance variation at 520 nm of **1-Glc** and **2-Glc** in PBS buffer for 50 min.

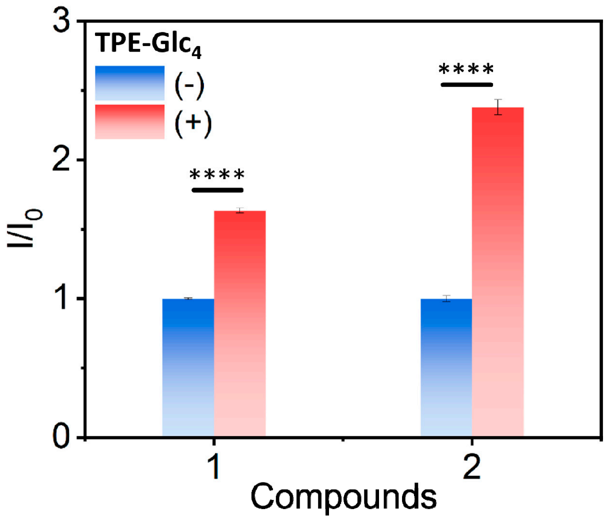

Figure S22. Ratio of quantitative fluorescence intensity in cells incubated with aggregates (blue bar) or glycol-dots (red bar) of **1** and **2**. I_0 is the original fluorescence intensity of aggregates in cells normalized as 1.

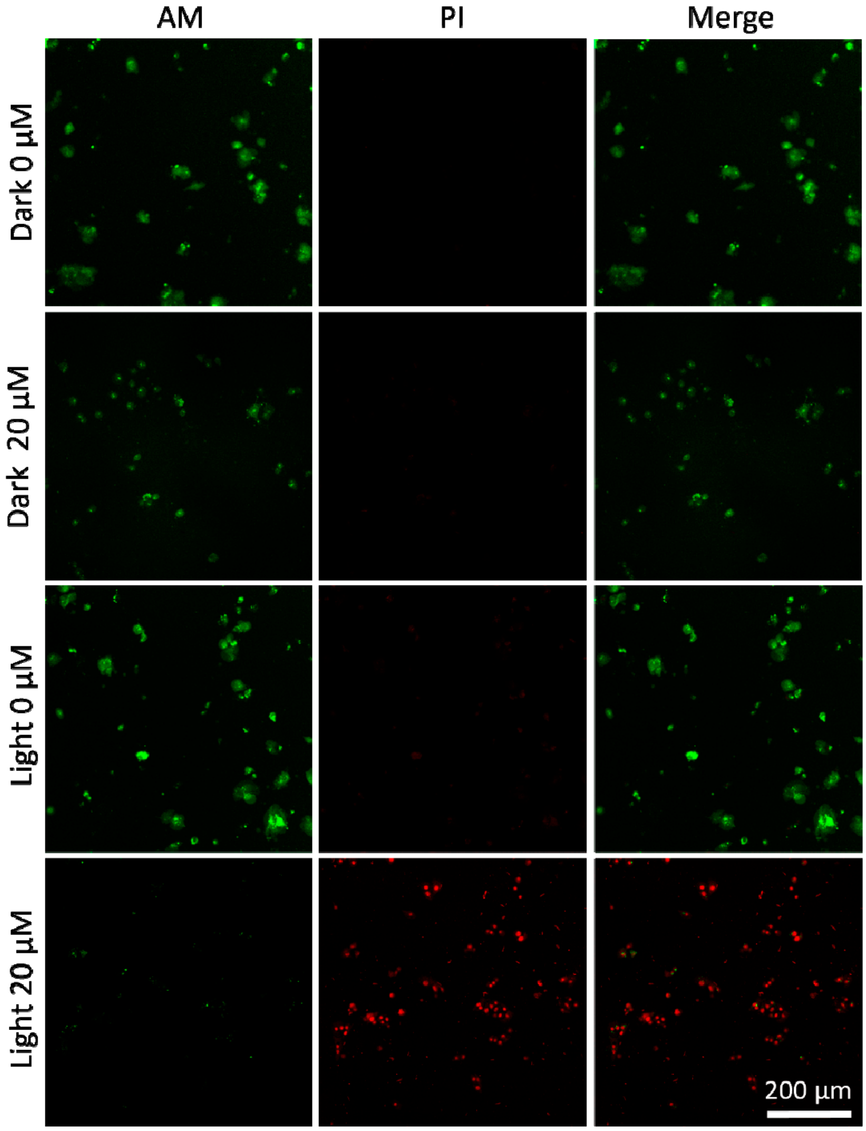

Figure S23. CLSM images of calcein AM (green) and propidium iodide PI (red) co-staining HepG2 cells for live-dead cells after treating with **2-Glc** (c = 20 µM) and irradiating with or without white LED light (P = 30 mW cm^{-2}, 2 h).

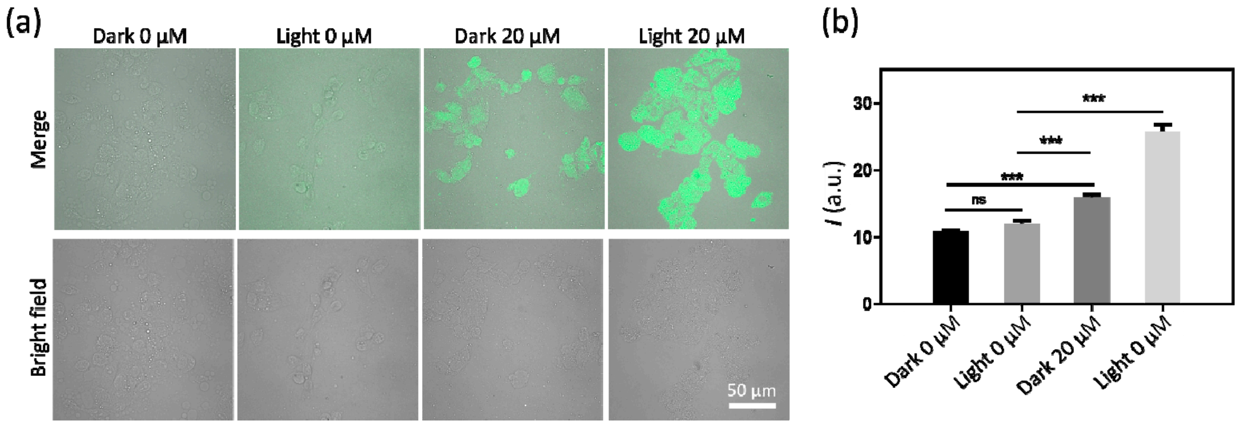

Figure S24. (a) The merge and brightfield images of DCFH-DA sensing the intracellular ROS generation after treating with **2-Glc** (c = 20 μM) and irradiating with or without white LED light (P = 30 mW cm^{-2}, 2 h). (b) Relative fluorescence intensity of DCFH-DA in each cell group after treating with **2-Glc** (c = 20 μM) and irradiating with or without white LED light.

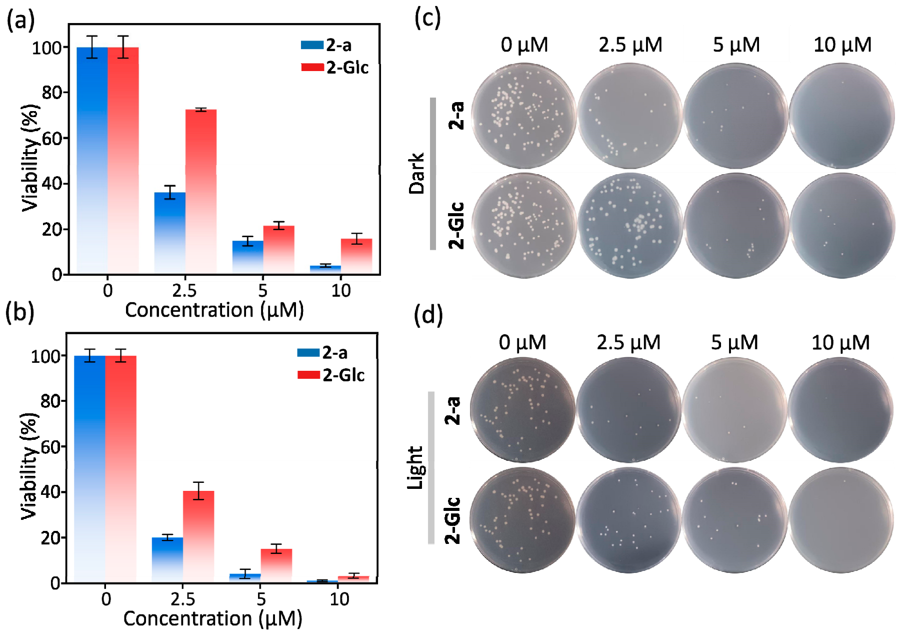

Figure S25. (a) Statistical relative activity of *E. coli* incubated with different concentrations of **2-a** and **2-Glc** (0-10 μM) in dark environment. (b) Statistical relative activity of *E. coli* incubated with different concentrations of **2-a** and **2-Glc** (0-10 μM) in light irradiation (**KL**: **TPE-Glc$_4$** = 1:5, c/c, P = 30 mW cm^{-2}, 2 h). (c) Plate images of *E. coli* incubated with different concentrations of **2-a** and **2-Glc** (0-10 μM) in dark environment. (d) Plate images of *E. coli* incubated with different concentrations of **2-a** and **2-Glc** (0-10 μM) in light irradiation (**KL**: **TPE-Glc$_4$** = 1:5, c/c, P = 30 mW cm^{-2}, 2 h).

^{1}H NMR and [13] NMR Spectra

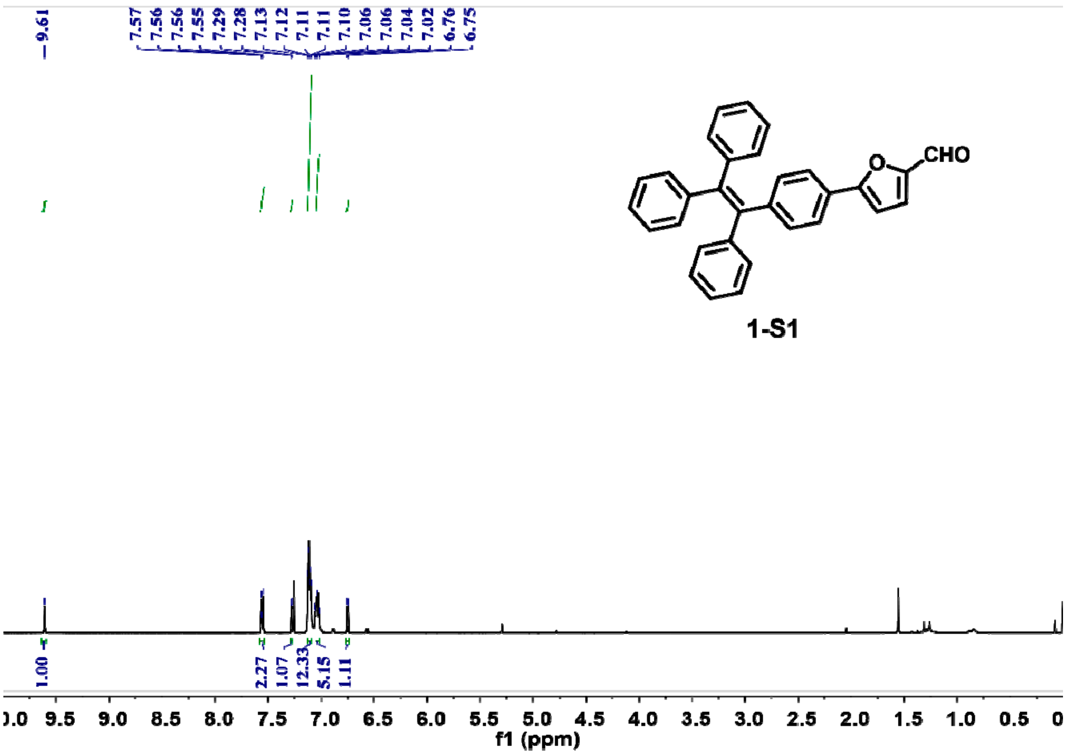

Figure S26. ^{1}H-NMR of compound **1-S1** in $CDCl_3$.

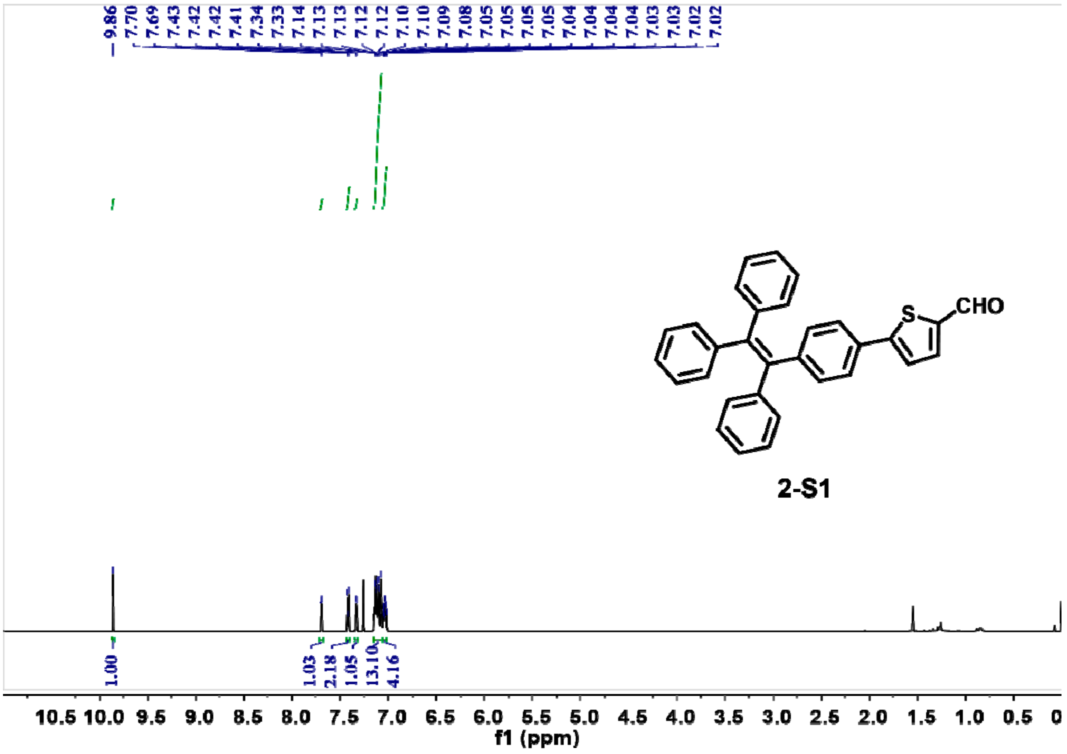

Figure S27. ^{1}H-NMR of compound **2-S1** in $CDCl_3$.

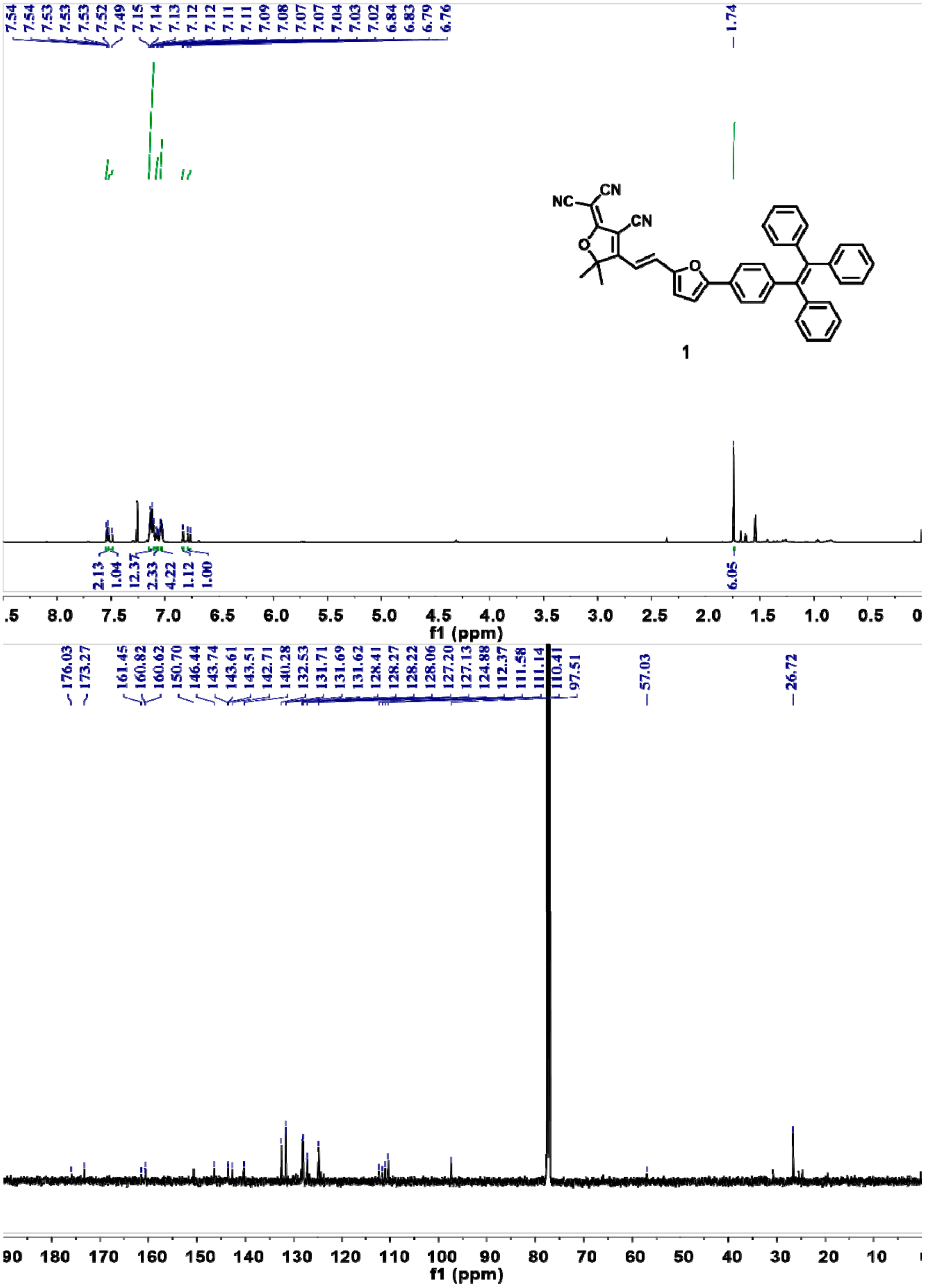

Figure S28. ^{1}H-NMR and ^{13}C-NMR of compound **1** in $CDCl_3$.

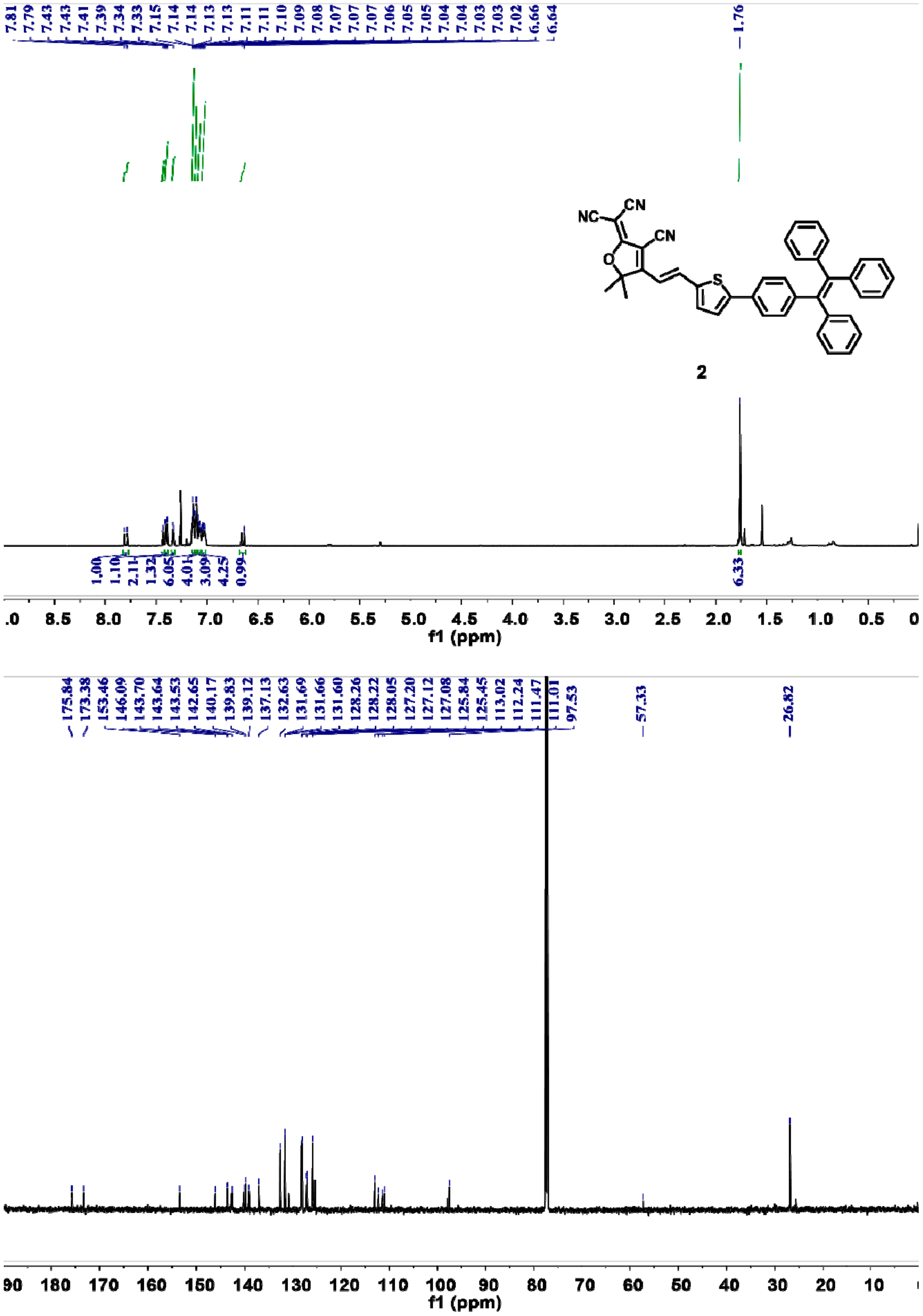

Figure S29. ^{1}H-NMR and ^{13}C-NMR of compound **2** in $CDCl_3$.

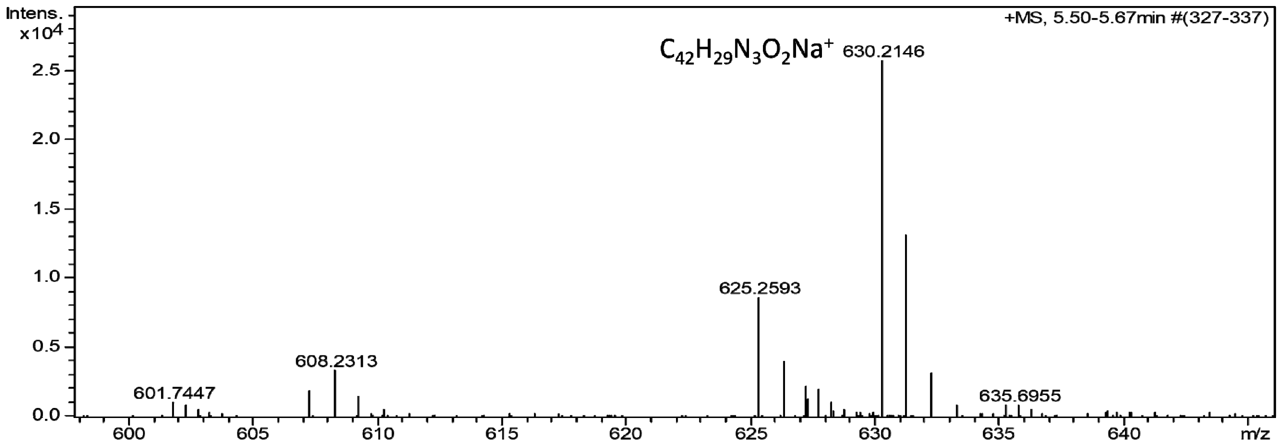

Figure S30. HRMS spectrum of **1**.

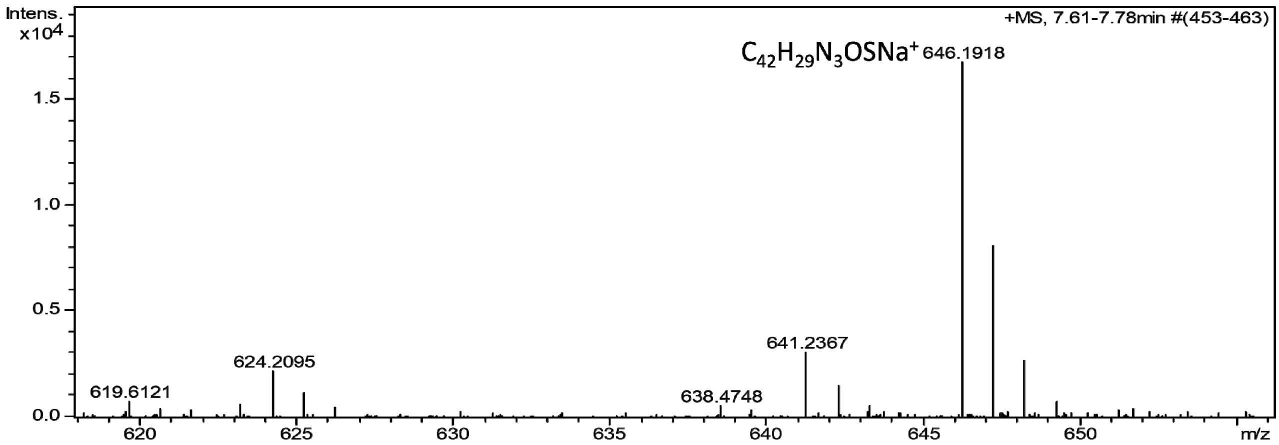

Figure S31. HRMS spectrum of **2**.

Reference

1. L. Dong, M. Fu, L. Liu, H.-H. Han, Y. Zang, G.-R. Chen, J. Li, X.-P. He and S. Vidal, *Chem. Eur. J.*, 2020, **26**, 14445-14452.
2. R. B. Gaussian 16, M. J. Frisch, G. W. Trucks, H. B. Schlegel, G. E. Scuseria, M. A. Robb, J. R. Cheeseman, G. Scalmani, V. Barone, G. A. Petersson, H. Nakatsuji, X. Li, M. Caricato, A. V. Marenich, J. Bloino, B. G. Janesko, R. Gomperts, B. Mennucci, H. P. Hratchian, J. V. Ortiz, A. F. Izmaylov, J. L. Sonnenberg, D. Williams-Young, F. Ding, F. Lipparini, F. Egidi, J. Goings, B. Peng, A. Petrone, T. Henderson, D. Ranasinghe, V. G. Zakrzewski, J. Gao, N. Rega, G. Zheng, W. Liang, M. Hada, M. Ehara, K. Toyota, R. Fukuda, J. Hasegawa, M. Ishida, T. Nakajima, Y. Honda, O. Kitao, H. Nakai, T. Vreven, K. Throssell, J. A. Montgomery, Jr., J. E. Peralta, F. Ogliaro, M. J. Bearpark, J. J. Heyd, E. N. Brothers, K. N. Kudin, V. N. Staroverov, T. A. Keith, R. Kobayashi, J. Normand, K. Raghavachari, A. P. Rendell, J. C. Burant, S. S. Iyengar, J. Tomasi, M. Cossi, J. M. Millam, M. Klene, C. Adamo, R. Cammi, J. W. Ochterski, R. L. Martin, K. Morokuma, O. Farkas, J. B. Foresman, and D. J. Fox, Gaussian, Inc., Wallingford CT, 2016.
3. T. Lu and F. Chen, *J. Comput. Chem.*, 2012, **33**, 580-592.
4. Z. Liu, T. Lu and Q. Chen, *Carbon*, 2020, **165**, 461-467.
5. T. Lu, *J. Chem. Phys.*, 2024, **161**, 802503.
